# Determining potential therapeutic targets for venous thrombosis through network pharmacology, cluster typing, WGCNA, machine learning, immune infiltration analysis, and ceRNA networks

**DOI:** 10.1097/MD.0000000000043018

**Published:** 2025-11-28

**Authors:** Zhuoji Li, Ming Zhong, Kaili Fu, Lingpin Pang, Jie Sun, Tao Huang, Lingyue Song, Dingyu Guo, Junfen Cheng, Xishi Sun

**Affiliations:** a The Second Affiliated Hospital of Guangdong Medical University, Zhanjiang, Guangdong Province, China; b Affiliated Hospital of Guangdong Medical University, Zhanjiang, Guangdong Province, China.

**Keywords:** ceRNA networks, cluster typing, immune infiltration analysis, machine learning, venous thrombosis, WGCNA

## Abstract

Venous thrombosis presents a significant global health challenge, characterized by its high incidence and limited therapeutic effectiveness. Our objective is to leverage the pharmacological insights offered by traditional Chinese medicine networks to identify potential therapeutic targets for venous thrombosis treatment and elucidate their underlying mechanisms of action. We initiated our study by isolating the active ingredients and targets of Chinese medicine compounds from the traditional Chinese medicine systems pharmacology database. Concurrently, we acquired venous thromboembolism (VTE) patient data from the gene expression omnibus dataset. Utilizing cytoscape, we constructed a network connecting traditional Chinese medicine ingredients, disease targets, and related interactions. Molecular subtypes were determined through target information clustering and typing using the “ConsensusClusterPlus” software package. Key genes were subsequently identified through a combination of weighted gene co-expression network analysis and machine learning techniques. Functional enrichment analysis was performed on these key genes. Subsequently, we investigated immune cell and immune function differences related to the identified key genes. Lastly, we constructed the competing endogenous RNA network associated with PPP2R1A. Our initial analysis identified several key genes, including FOS, ICAM1, CASP3, and HSP90AA1. Subsequent functional and downstream analyses revealed that 2 central hub genes, PPP2R1A and ribosomal protein L9, may represent novel targets for VTE therapy. Notably, these hub genes are not only associated with thrombospondin and platelet function regulation but also play a role in modulating T cell expression in immunoassays. In conclusion, our findings suggest that 10 long non-coding RNAs may compete with 2 microRNAs for binding, thereby regulating PPP2R1A target genes, with potential implications for improving VTE treatment efficacy. Our current findings offer a novel direction and serve as a theoretical foundation for identifying therapeutic targets in the treatment of VTE. Furthermore, these findings hold the potential to facilitate the translation of our research into clinical applications in the future.

## 1. Introduction

Venous thromboembolism (VTE), the third most common cardiovascular disease globally, includes deep vein thrombosis (DVT) and pulmonary embolism (PE). It often presents with leg pain, chest discomfort, and shortness of breath, and can be fatal. In the United States, there are about one million cases annually, resulting in around 300,000 deaths. Nearly two-thirds of recurrent VTE events are linked to hospitalization.^[1]^ This highlights the high risk of developing VTE and the poor prognosis associated with it. The treatment of venous thromboembolism requires immediate anticoagulant therapy tailored to individual bleeding risk assessments. In recent years, direct oral anticoagulants have become an important choice for patients, as reflected in the latest treatment guidelines from the American College of Chest Physicians.^[2]^

Treatment for VTE includes anticoagulation, thrombolysis, thrombectomy, balloon angioplasty or stent placement, and compression therapy, among others. In China and some other countries, traditional Chinese medicine (TCM) has been used for over 2000 years. TCM offers advantages in treating VTE through holistic regulation, fewer side effects, multi-target effects, and personalized treatment. Additionally, network pharmacology further elucidates the complex mechanisms of TCM formulations, enhancing our understanding of their therapeutic benefits.

In TCM, venous thrombosis is associated with blood stasis, diagnosed by assessing dampness, heat, stasis, and deficiency. Treatment focuses on nourishing qi and promoting blood circulation. Zhi Shi Xie Bai Gui Zhi Tang, a classic formula for PE, includes Gui Zhi (cinnamon twig) for improving blood flow.^[3]^ Belamcanda chinensis, known for promoting blood circulation and its anti-inflammatory properties, is used as the second ingredient.^[4]^ Safflower, effective in resolving blood stasis, is the third ingredient.^[5]^ Astragalus treats venous thrombosis and is used in Huangqi Guizhi Wuwu Tang, a formula used for nearly 2000 years.^[6]^ Ginseng, with ginsenosides Rb and Rg3, acts as an anticoagulant and inhibits platelet aggregation.^[7,8]^ The combination of these 5 herbs is called compound Chinese medicine (CCM). There is a lack of systematic studies on CCM’s mechanisms in treating VTE, including target analysis, biological processes, and metabolic pathways.

TCM involves intricate compounds with diverse pathological targets and pathways, which pose limitations to conventional pharmacological methods for studying TCM mechanisms.^[9]^ In 2013, Shao Li introduced the novel concept of “network pharmacology,” offering a fresh perspective for elucidating the mechanisms of action behind TCM formulas.^[10]^ The realm of network pharmacology in TCM encompasses virtual computation, high-throughput data analysis, and network database searching, involving the construction of bioinformatics networks and the subsequent analysis of network topology.^[11]^ This approach centers on the synergy of multiple components, channels, and objectives, making it particularly well-suited for TCM analysis.^[12,13]^ In recent years, the integration of bioinformatics and computational prediction-based network pharmacology has emerged as a robust method to systematically uncover the molecular-level biological mechanisms underlying complex diseases and drug actions.^[14,15]^

Cyberpharmacology is increasingly employed to investigate the mechanisms of CCM in the treatment of various diseases.^[16,17]^ In our study, we harnessed network pharmacology to elucidate the effects of CCM on VTE. Through meticulous network pharmacology analysis, we initially pinpointed critical target intersections. We then conducted clustering analysis and applied the weighted gene co-expression network analysis (WGCNA) method, intersecting the outcomes with gene expression matrix data for VTE. This process enabled us to identify key genes. Subsequently, we employed machine learning techniques, including the Random Forest and Lasso algorithms, to further refine the selection of these crucial genes.Once we had determined the key genes, we conducted comprehensive examinations of their functional pathways. This included in-depth immune cell differential analysis, immune function differential analysis, and the generation of receiver operating characteristic (ROC) curves for the genes, among other assessments, to evaluate their efficacy. Finally, we constructed a network of competing endogenous RNAs (ceRNAs) associated with the PPP2R1A gene through gene enrichment analysis and target gene prediction. Within this network, we identified 2 microRNAs (miRNAs) and ten significant long noncoding RNAs (lncRNAs), providing valuable insights for subsequent research and the development of treatments for vascular thromboembolism.

This study utilizes a network pharmacology approach to systematically predict the multifaceted actions of CCM in treating venous embolism. By constructing a comprehensive network model, we provide a theoretical foundation for further investigation into the pharmacodynamic components and mechanisms of CCM. Additionally, this research identifies novel molecular therapeutic targets and employs various analytical methods to explore specific biological issues, including genes, molecular pathways, and network relationships. Ultimately, this enhances our understanding of venous embolism mechanisms and diversifies treatment approaches.

## 2. Methods

### 2.1. Data preprocessing

We obtained the venous embolism dataset GSE19151 from Gene Expression Omnibus, comprising gene expression profiles from 70 patients with VTE and 63 healthy controls on the GPL571 platform. To identify relevant compounds, we queried the Traditional Chinese Medicine Systems Pharmacology Database and Analysis Platform (TCMSP) for ginseng, radix et rhizoma, cinnamon sticks, safflower, and astragalus. Subsequently, we analyzed the gene expression profiles of these 5 Chinese medicines, applying selection criteria of oral bioavailability (OB) ≥ 30% and drug-like properties (DL) ≥ 30%. We conducted preliminary screening of active ingredients based on values meeting the criteria of OB ≥ 30% and DL activity ≥ 0.18, which are indicative of attributes related to absorption, distribution, metabolism, and excretion in vivo.

### 2.2. Variance analysis

We conducted differential expression analysis between the disease and healthy groups in the GSE19151 venous embolism dataset using the R package limma. Genes with a significance level (*P*-value) of <.05 were identified as differentially expressed genes.

### 2.3. Screening of drug-related targets

Retrieve the predicted target names of active ingredients for drugs from the TCMSP database. Compile the acquired target names for each drug into a unified table. Translate protein targets of active ingredients into canonical IDs using the UniProt database. Identify common targets between drug-specific active targets and disease-related targets.

### 2.4. Protein–protein interaction network construction of compound drug components and venous thrombosis disease targets

To comprehensively elucidate the interactions between compound drug-related targets and venous embolism disease targets, we utilized the STRING database to construct a protein–protein interaction (PPI) network of potential targets specifically related to PE. Disease targets were identified through differential gene screening.

Subsequently, we created a network that connects herbal medicine components with disease targets using Cytoscape 3.8.0. In this network, we also identified hub genes.

### 2.5. Functional enrichment analysis

Gene Ontology enrichment analysis of intersecting target genes was conducted using the DAVID database. The top 6 results with the smallest *P*-values were selected for visualization. Kyoto Encyclopedia of Genes and Genomes (KEGG) enrichment analysis of intersecting target genes was performed using the clusterProfiler R package. Significant enrichment results with *P*-values < .05 were chosen, and the top 20 results were visualized. Network diagrams for KEGG were constructed using Cytoscape 3.8.0.

### 2.6. Clustering and typing

Intersecting target genes underwent cluster analysis utilizing the “ConsensusClusterPlus” software package, aiming to unveil subtypes linked to these intersecting target genes. A heatmap was employed to illustrate the correlations between the clusters and the genes, while a box plot was employed to display the expression variations of genes within the clustered subtypes.

### 2.7. Weighted gene co-expression network analysis

We utilized the “WGCNA” package to identify key genes significantly associated with both the clustered subtypes and the venous embolism score. To achieve this, we employed the expression profiles of the top 25% variants from the venous embolism samples and the top 25% variants from the typing results files as input data.

Subsequently, we determined the soft threshold, conducted clustering of the neighbor matrix, and pinpointed hub modules. By calculating Pearson correlation coefficients between these modules and scores, we identified the modules with the strongest positive correlations for further analysis. We then performed an intersection of genes within the 2 modules, as obtained from the 2 WGCNA analyses.

### 2.8. Construction and validation of predictive features

The genes obtained from the intersection were initially subjected to preliminary analysis using a decision tree. Further analysis involved employing lasso regression, random forest, and support vector machine (SVM) algorithms to identify key genes and construct a set of disease-related genes. The genes identified from these 3 algorithms were then intersected.

To validate the results, ROC analysis was conducted. Finally, the genes resulting from this intersection were utilized to assess differences in expression through line graphs and violin plots in the sample files.

### 2.9. Feature-based gene set enrichment analysis

We employed gene set enrichment analysis (GSEA) to investigate the potential pathways and functional mechanisms associated with the genes. The reference gene sets utilized included c5.go.Hs.symbols.gmt and c2.cp.kegg.Hs.symbols.gmt.

For filtering, we applied the following criteria: normalized enrichment score > 1, nominal output measurement *P*-value < .05, and false discovery rate *q*-value < 0.25.

### 2.10. Feature-based gene set variation analysis

We applied gene set variation analysis (GSVA) to investigate potential pathways of action and mechanistic functions of genes. The reference gene sets included c5.go.Hs.symbols.gmt and c2.cp.kegg.Hs.symbols.gmt. We applied a screening condition of *P*-value < .05, and the results were visualized using a histogram.

### 2.11. Immune landscape analysis

We utilized the IMMPORT tool to download immune gene set files. Subsequently, we categorized samples into high and low-risk groups based on target genes. To assess differences in immune-related function between these groups, we employed the ssgsea immune correlation algorithm.

We conducted an analysis of immune cell infiltration using the CIBERSORT algorithm, aiming to investigate correlations between immune cells and expression differences between the venous embolism and healthy groups. For further analysis, we examined whether individual immune cells correlated with the resultant genes (*P*-value < .05) from the immune infiltration files and visualized the results.

### 2.12. Construction of the ceRNA regulatory network

We predicted miRNAs and lncRNAs associated with PPP2R1A by utilizing miRanda, miRDB, and the TargetScan database. Following the ceRNA hypothesis, we constructed a ceRNA network and visualized the results using Cytoscape.

## 3. Results

### 3.1. Acquisition of active ingredients of compound drugs

We retrieved data from the TCMSP database, focusing on 5 herbs: ginseng, astragalus, cinnamon sticks, radix et rhizoma, and safflower. These herbs were screened based on criteria of OB ≥ 30% and DL ≥ 0.18 to identify potentially active ingredients. Our analysis revealed 23 potent ingredients in ginseng, 87 in astragalus, 7 in cinnamon sticks, 8 in radix et rhizoma, and 5 in safflower.

### 3.2. Target prediction of compound drugs for venous embolization

We utilized the TCMSP database, primarily drawing data from DrugBank, to identify relevant targets for each drug. Gui Zhi had 2402 active targets, Safflower had 1466 active targets, Astragalus had 953 targets, Ginseng had 748 targets, and Saganum had 736 targets. All active ingredient targets were consolidated into a comprehensive table, resulting in 1968 targets. These target names were standardized for consistency.

**Figure 1.**
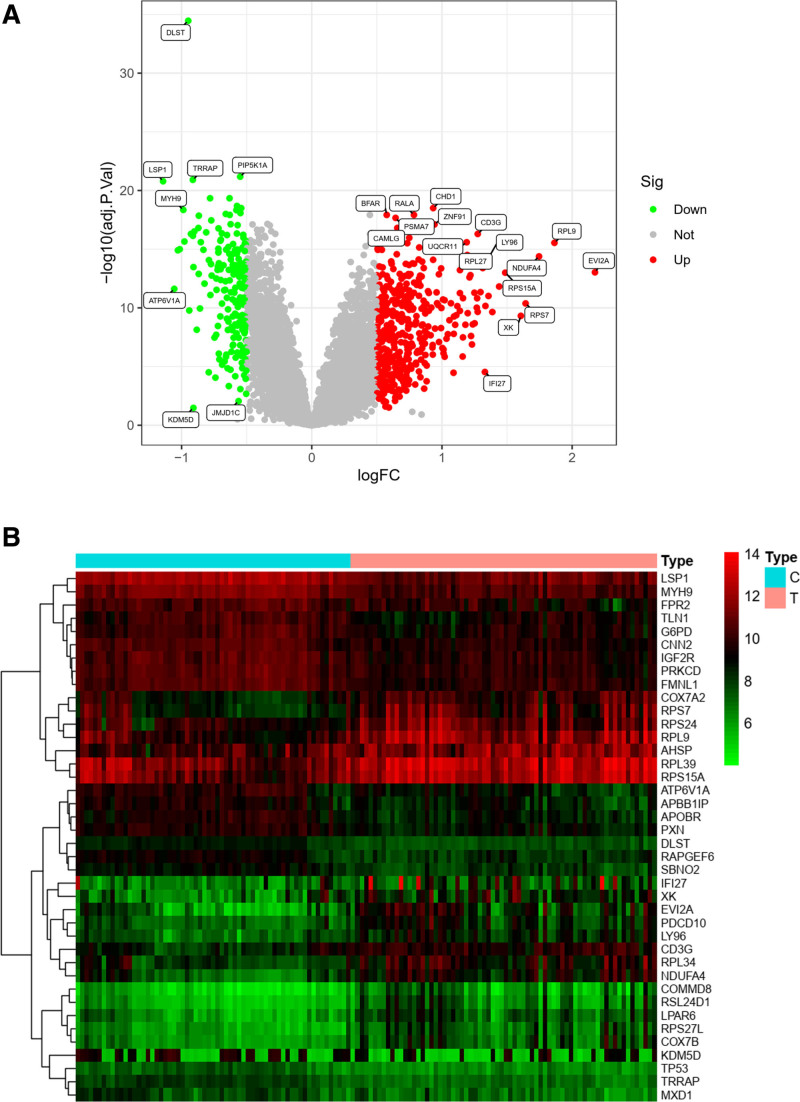
Differential analysis of genes (volcano and clustered heat maps). (A) A volcano plot of the VTE matrix, with red dots representing up-regulated expression and green color representing down-regulated expression; (B) represents a clustered heat map of the VTE matrix. The top 40 genes with significant differences are shown. The top type is the disease group and the healthy group, and the lower red bar shows up-regulated expression, while the green bar shows down-regulated expression. VTE = venous thromboembolism.

In conjunction with the gene expression omnibus database, we acquired gene expression profiles from 70 patients with VTE and 63 healthy controls. We conducted differential gene expression analysis using the limma R package to identify genes potentially associated with venous embolism. This analysis yielded a total of 627 significantly different genes in patients with venous embolism compared to controls, comprising 430 up-regulated genes and 197 down-regulated genes. Visualization of all gene expressions in the dataset was achieved through volcano plots and clustered heat maps (Fig. 1).

To establish potential therapeutic targets for venous embolism through compound drugs, we compared and matched the potential active ingredient targets with the disease targets. Ultimately, we identified 14 potential targets associated with the treatment of venous embolism.

### 3.3. Construction of compound drug-vein embolism target PPI network

The targets of active ingredients in the screened compound drugs were compared and intersected with the targets associated with venous embolism. This analysis yielded 14 common targets shared between the compound drugs and venous embolism.

**Figure 2.**
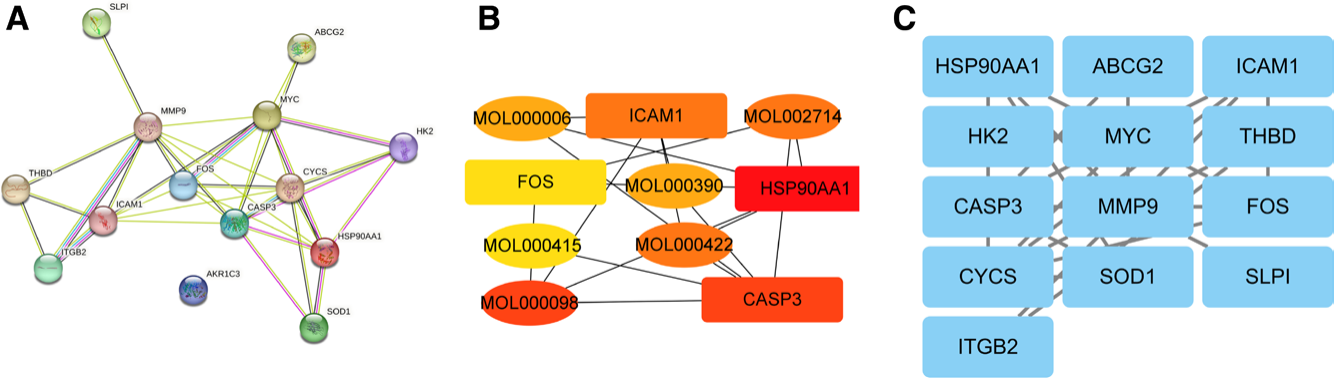
Construction of PPI network of compound drug-vein embolization targets. (A) The PPI network of DE-VTE based on the STRING database. (B) Five hub genes obtained from the “cytohubba” plug-in. (C) 13 modular genes obtained from the “MCODE” plug-in. PPI = protein–protein interaction, VTE = venous thromboembolism.

Subsequently, these targets were input into the STRING 11.0 platform (https://string-db.org) to generate a PPI network (Fig. 2A). Using the cytoHubb plugin in Cytoscape, we identified key genes within this network. The top 5 hub genes were determined based on their degree centrality (Fig. 2B). Additionally, we identified 13 module genes using the MCODE module (Fig. 2C).

### 3.4. Enrichment analysis of target functions and pathways

We conducted gene ontology enrichment analysis of the intersecting target genes using the DAVID database and selected the top 6 results with the smallest *P*-values for visualization. Additionally, we performed KEGG enrichment analysis of these intersecting target genes using the clusterProfiler R package, focusing on significant enrichment results with *P*-values < .05. We then visualized the top 20 results.

**Figure 3.**
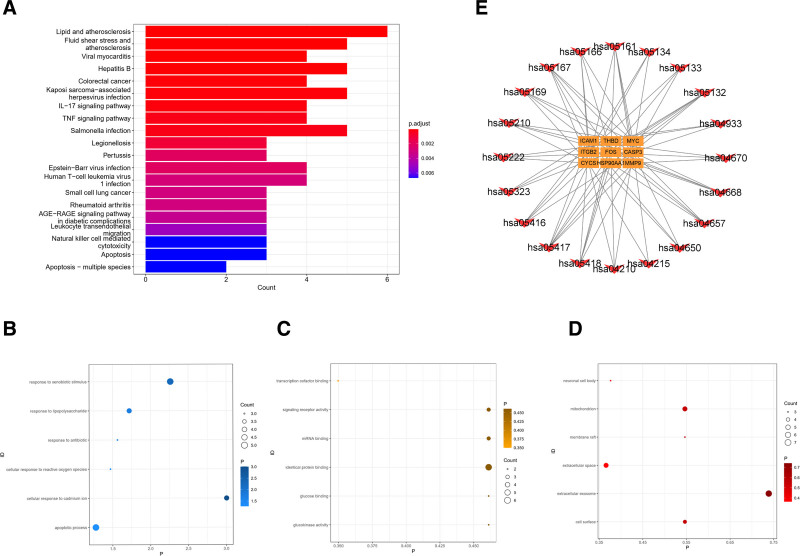
Enrichment analysis. (A) A bar graph of KEGG enrichment pathway, vertical coordinate is the name of KEGG, horizontal coordinate is the proportion of enriched genes, and the size of the circle is the number of genes enriched, and the redder the color is, the more significant the enrichment is. (B) Bubble plot of BP bioprocesses in GO enrichment analysis showing the first 6 enrichment results, vertical coordinate is the name of GO, horizontal coordinate is the number of genes, and darker color means more significant enrichment results. (C) Bubble plot of MF molecular function in GO enrichment analysis, showing the first 6 enrichment results, vertical coordinate is GO name, horizontal coordinate is gene number, and darker color indicates more significant enrichment results. (D) Bubble plot of CC cellular components in GO enrichment analysis showing the first 6 enrichment results, vertical coordinates are GO names, horizontal coordinates are gene numbers, and darker colors indicate more significant enrichment results. (E) A KEGG network graph with red arrow nodes representing enrichment pathways and yellow rectangular nodes representing genes. GO = gene ontology, KEGG = Kyoto Encyclopedia of Genes and Genomes.

The results were presented through bubble plots and bar graphs, revealing that the pathways associated with the compound drug-vein embolization targets primarily included lipid and atherosclerosis, fluid shear stress and atherosclerosis, myocarditis, hepatitis B, colorectal cancer, among others (Fig. 3A). Biological processes encompassed cellular responses to cadmium ions, xenobiotic stimuli, lipopolysaccharides, antibiotics, reactive oxygen species, as well as apoptotic processes (Fig. 3B). Molecular functions involved identical protein binding, glucokinase activity, glucose binding, signaling receptor activity, messenger ribonucleic acid binding, and transcriptional cofactor binding (Fig. 3C). Cellular components comprised extracellular exosomes, cell surfaces, membrane rafts, mitochondria, neuronal cell bodies, and extracellular spaces (Fig. 3D).

Furthermore, we constructed a KEGG relationship network diagram to illustrate the interconnected relationships between KEGG pathways and key enriched genes (Fig. 3E).

### 3.5. Construction of target-pathway network of compound drug for venous embolism treatment

**Figure 4.**
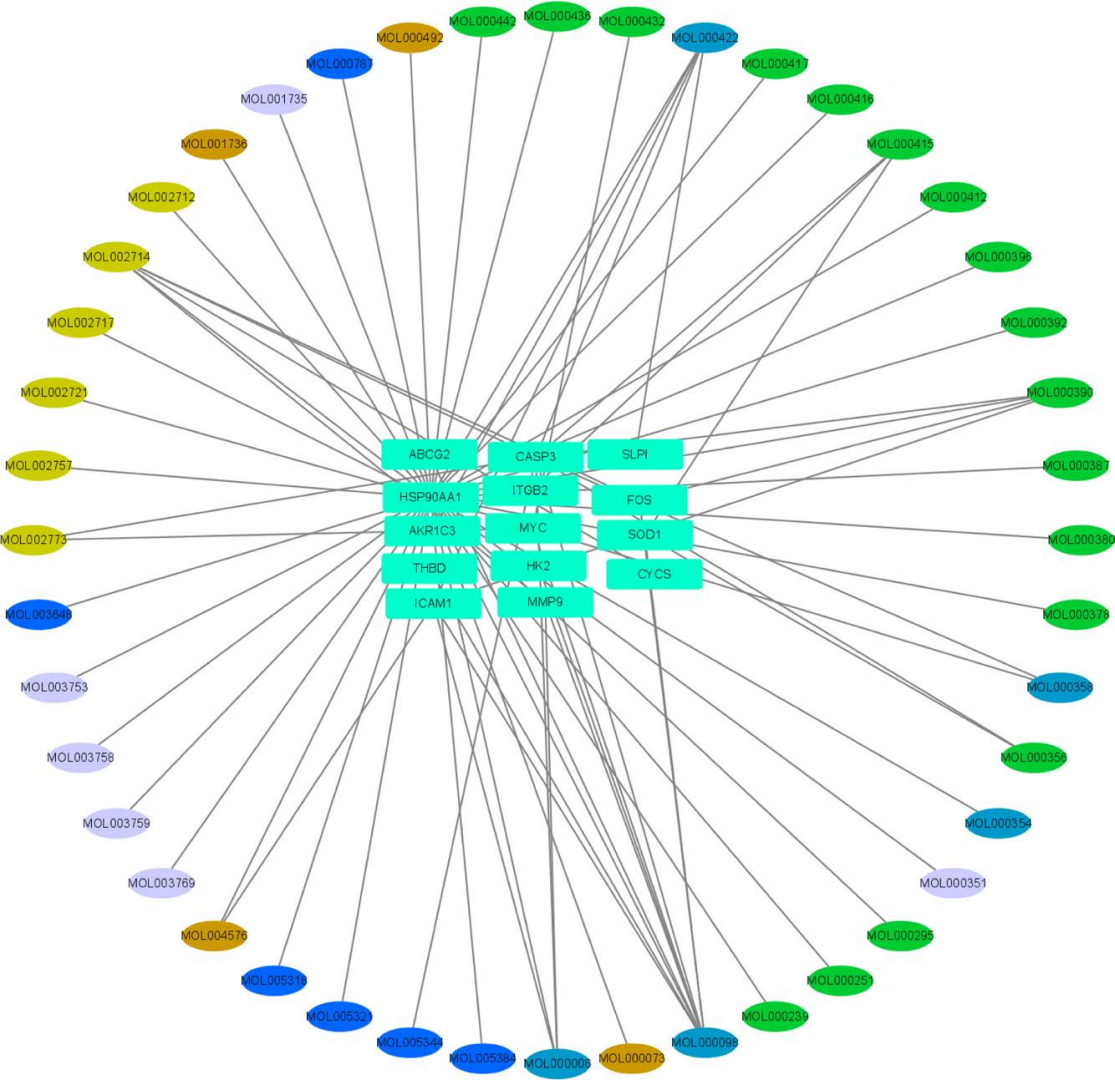
Compound drug composition-vein embolization target-pathway network map. The compound drug composition-vein embolization target-pathway network, the oval nodes are the active ingredients of the drug, the rectangle is the gene target, the light yellow is the safflower active ingredient, the brown is the cinnamon branch active ingredient, the green is the astragalus active ingredient, the blue is the ginseng active ingredient, and the purple is the Sagittaria active ingredient.

We utilized Cytoscape 3.8.0 to construct the target-pathway network for venous embolism, as depicted in Fig. 4. In this figure, oval nodes represent the active ingredients of the drug, rectangular nodes represent target points, and the right side of the figure illustrates the regulatory network. Safflower is represented in light yellow, cinnamon stick in brown, astragalus in green, ginseng in blue, and saikan in purple.

The results of the Cytoscape network analysis revealed a complex relationship between the ingredients and the targets. This complexity included “one-to-many,” where one ingredient acted on multiple targets, “many-to-one,” and “many-to-many” interactions. The Cytoscape network analysis underscored the intricate relationship between components and targets, characterized by “one-to-many,” where one component affected multiple targets, and “many-to-one,” where multiple components influenced a single target, thereby participating in the regulation of the pathway. These findings highlight the “multi-component, multi-target, multi-pathway” nature of compound drug treatments for venous embolism.

### 3.6. Plotting the positional circles of each gene on the chromosome and correlation matrix analysis of genes

**Figure 5.**
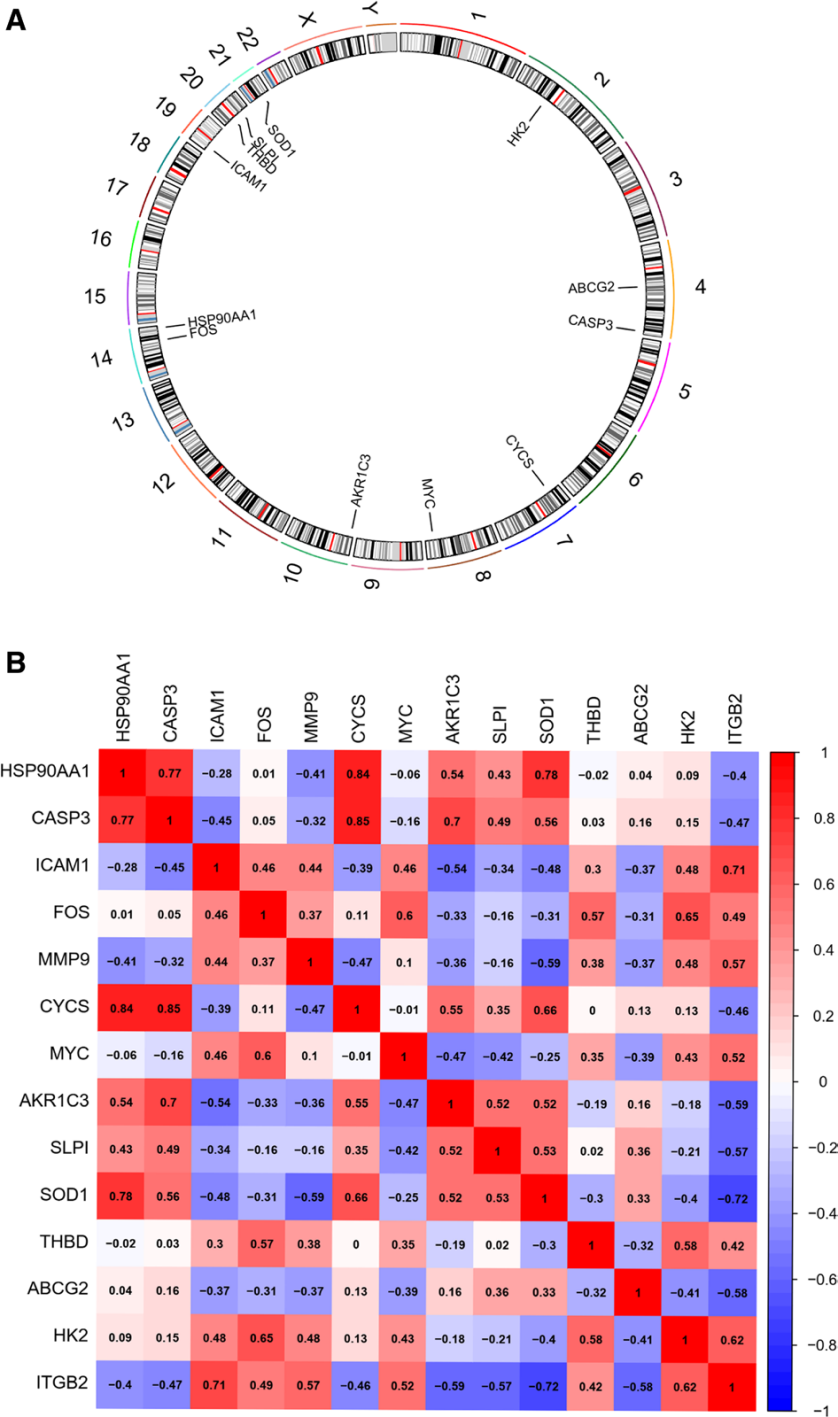
Plotting the positional circles of individual genes on chromosomes and correlation matrix analysis of genes. (A) A gene chromosome circle diagram that shows the location of each gene on the chromosome. (B) The genetic correlation of each gene, red is positively correlated regulation and blue is negatively correlated regulation, the darker the color indicates the stronger the correlation.

We employed chromosome and gene location files to create positional maps illustrating the locations of each gene on the chromosome (Fig. 5A). Additionally, we visualized the correlations between genes using the corrplotR package. In the correlation plot (Fig. 5B), stronger positive correlations are represented by darker shades of red, while stronger negative correlations are indicated by darker shades of blue (or green).

### 3.7. Clustering typing and scoring co-expression networks

**Figure 6.**
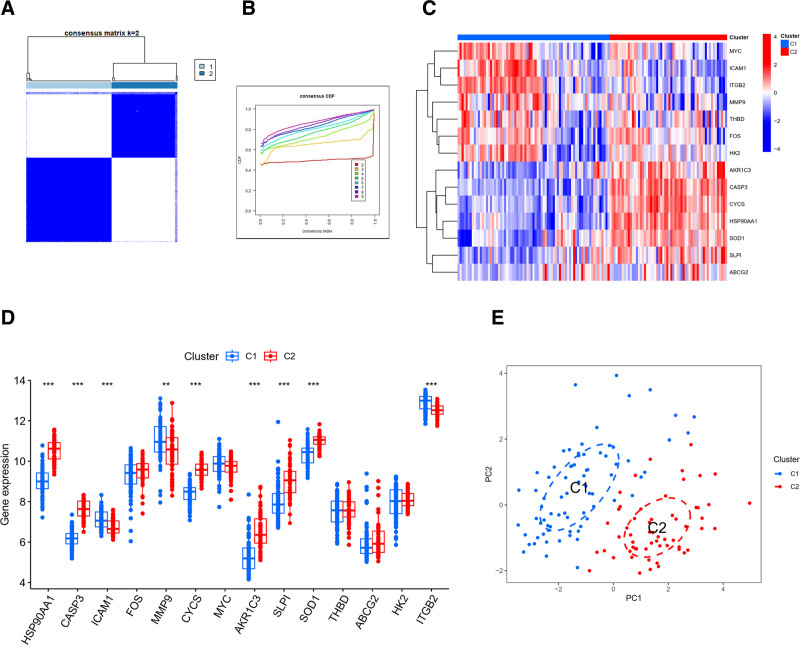
Clustered typing and typing heat map. (A) The consensus clustering matrix for k = 2. (B) The CDF of consensus clustering for k values of 2 to 9. (C) A heat map of fourteen genes between 2 clusters. (D) A box line plot of expression differences for fourteen genes between 2 clusters. (E) The PCA plot between the 2 clusters, indicating that there is a significant clustering effect with hierarchy.

We achieved optimal clustering results by dividing the obtained genes into 2 subgroups characterized by improved internal consistency and subgroup stability (Fig. 6A and B). Heatmaps (Fig. 6C) displayed the variations in gene expression between these 2 groups, while box plots (Fig. 6D) highlighted genes with significant expression differences.

Principal component analysis (PCA) plots illustrated the significant distinctions between the clusters (Fig. 6E).

**Figure 7.**
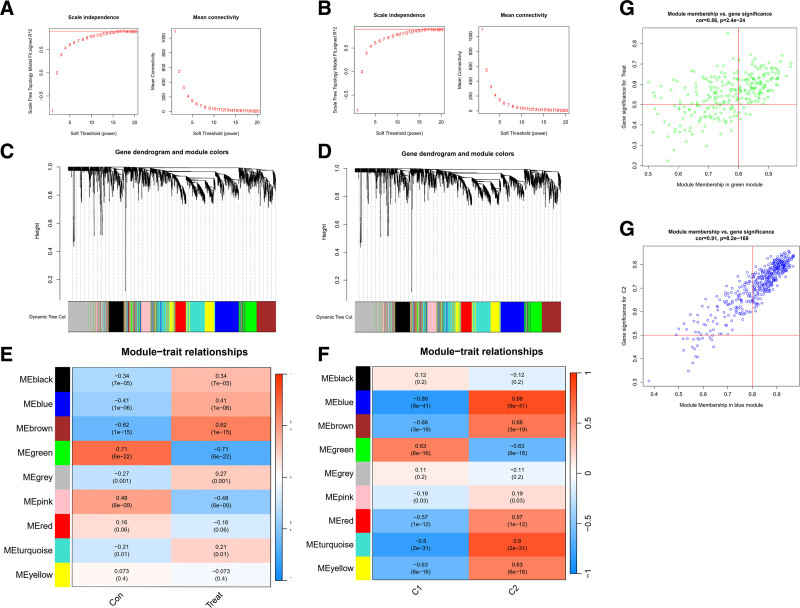
Scoring co-expression network diagram. (A) Scale independence and mean connectivity for the VTE cohort. (B) The scale independence and average connectivity of the typing cohort. (C) The gene dendrogram and modules of the VTE cohort before merging. (D) The gene dendrogram and modules of the typing cohort before merging. (E) The Pearson correlation analysis of merged modules in the VTE cohort. (F) The Pearson correlation analysis of merged modules in the typing cohort. (G) The scatter plot of green modules MM and GS in the VTE cohort. (H) A scatter plot of the blue modules MM and GS in the fractal cohort. GS = gene significance, MM = module membership, VTE = venous thromboembolism.

We conducted WGCNA using the expression profiles of the top 25% variance in both the typing cohort and the disease expression data cohort. A threshold of 15 was applied to both cohorts (Fig. 7A and B). Subsequently, dynamic module identification was carried out in different cohorts, each containing no <100 genes per module (Fig. 7C and D).

For the disease expression data cohort, we identified a total of 9 expression modules, with the green module displaying the strongest positive and negative correlations between con score (Cor = 0.71, *P* = 6E‐22) and treat score (Cor = ‐0.71, *P* = 6E‐22) (Fig. 7E). In the typing cohort, we identified 9 co-expression modules, with the blue module exhibiting the strongest positive and negative correlations for C1 score (Cor = ‐0.86, *P* = 9e‐41) and C2 score (Cor = 0.86, *P* = 9e‐41) (Fig. 7F).

We assessed the correlation between Gene-to-Module Membership and gene-trait correlation (gene significance), revealing positive correlations (Fig. 7G and H).

Finally, we identified potential venous embolism-associated genes by screening 632 genes in the blue module and 177 genes in the green module using thresholds of Module Membership > 0.6 and gene significance > 0.6.

### 3.8. Screening of key genes and their diagnostic efficacy

**Figure 8.**
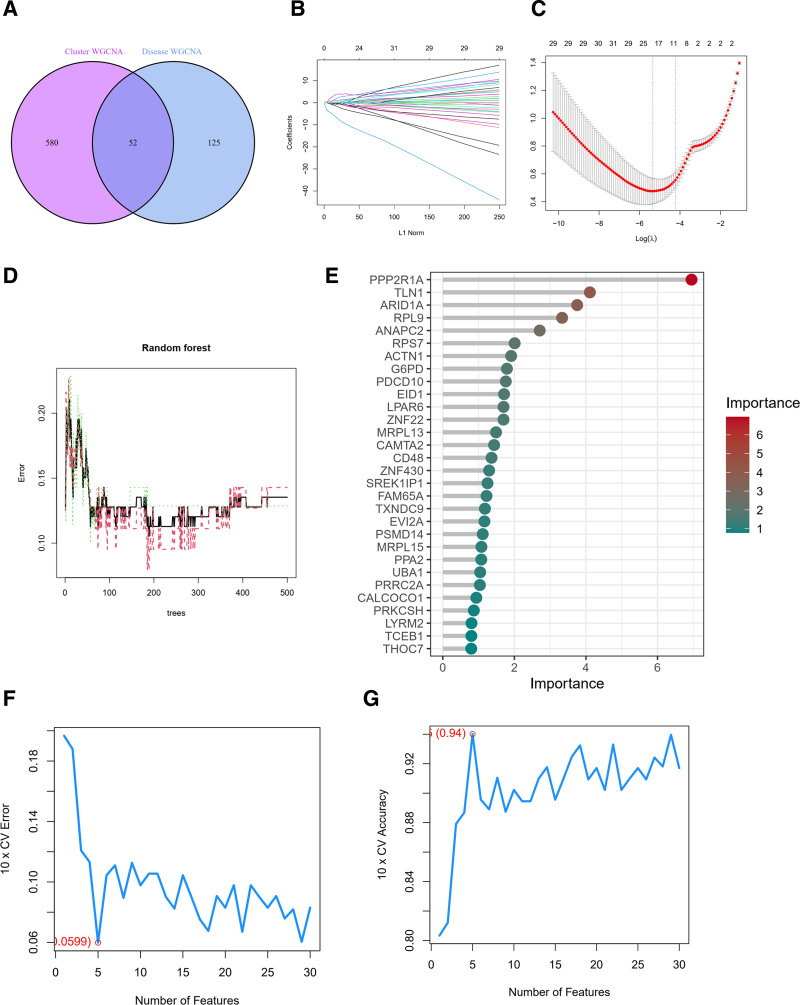
(A) The set of intersecting genes for the green module in the VTE cohort and the blue module in the typing cohort. (B) The lasso regression analysis, the process of selecting the best value of parameter λ in the Lasso regression model by the cross-validation method, and (C) the change characteristics of variable coefficients. (D) The error model of random forest, the red and green lines indicate the errors of the 2 categories of classification, respectively, and the black line indicates the average error; (E) the importance of each gene in the model. (F and G) The screening genes using the support vector machine recursive feature elimination (SVM-RFE) algorithm. VTE = venous thromboembolism.

We performed an intersection analysis of the correlated genes, resulting in the identification of 52 genes (Fig. 8A). Subsequently, employing Lasso regression analysis, we narrowed down the selection to 22 genes exhibiting high correlations (Fig. 8B and C). Random forest analysis was then applied to further refine the list, resulting in the identification of 6 genes with strong correlations (Fig. 8D and E). Subsequent SVM machine learning screening led to the identification of 5 genes characteristic of the disease (Fig. 8F and G).

**Figure 9.**
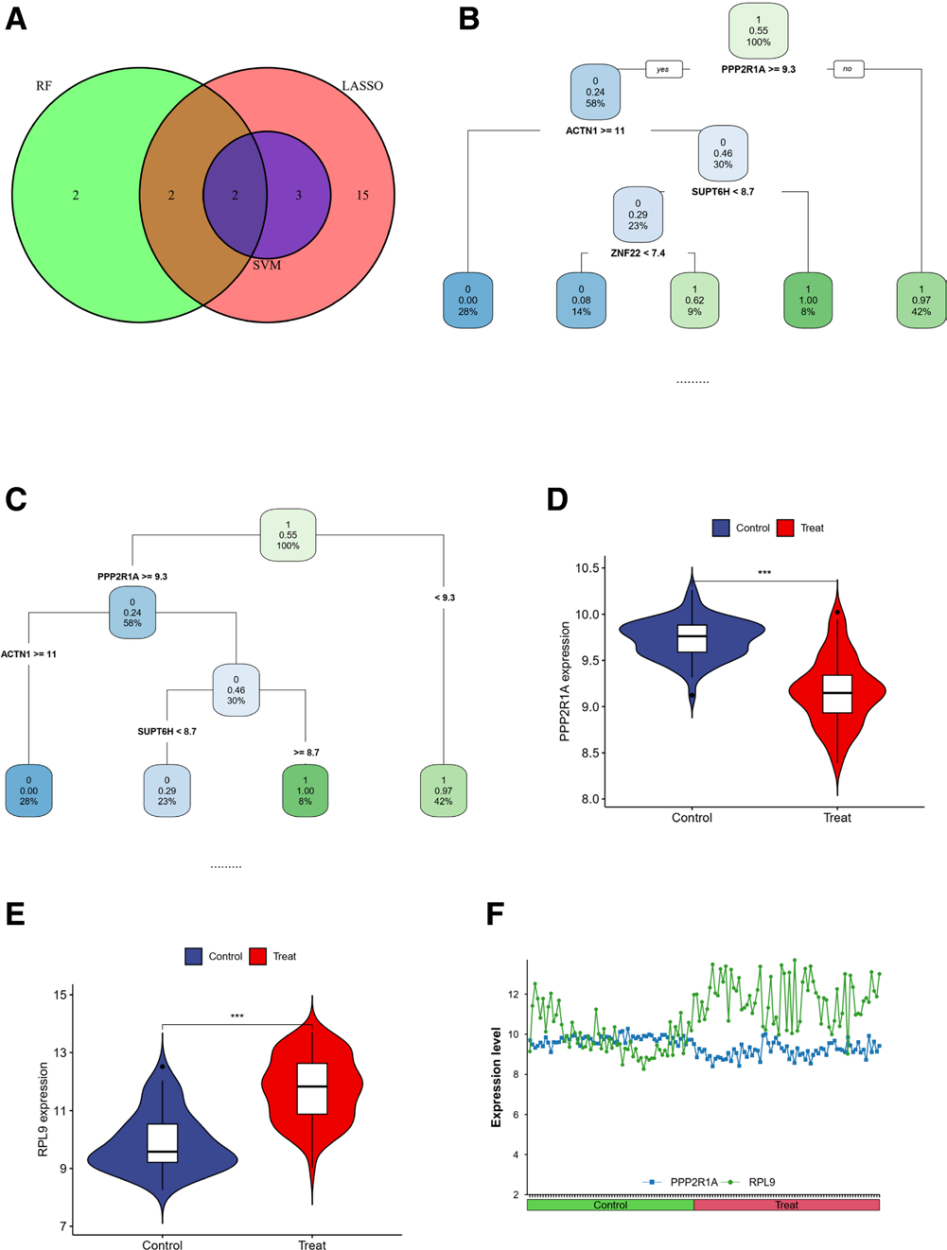
(A) The ensemble of genes obtained from the 3 screening methods. (B and C) Decision tree analyses showing PPP2R1A as the key gene in both pruned and unpruned rules. (D and E) The expression differences of 2 key genes between healthy and diseased groups. (F) A line graph, which shows that the difference in the expression of the 2 genes is more obvious in the disease group.

Next, we intersected the obtained genes, ultimately identifying PPP2R1A and ribosomal protein L9 (RPL9) as key genes (Fig. 9A). We further conducted decision tree analysis, considering both pruned and unpruned decision tree results, which consistently highlighted PPP2R1A as the key gene (Fig. 9B and C).

To visualize the significant expression differences between healthy and diseased groups, we employed violin plots for both genes (Fig. 9D and E). Fold change plots indicated that the expression difference between the 2 genes was more pronounced in the disease group (Fig. 9F).

**Figure 10.**
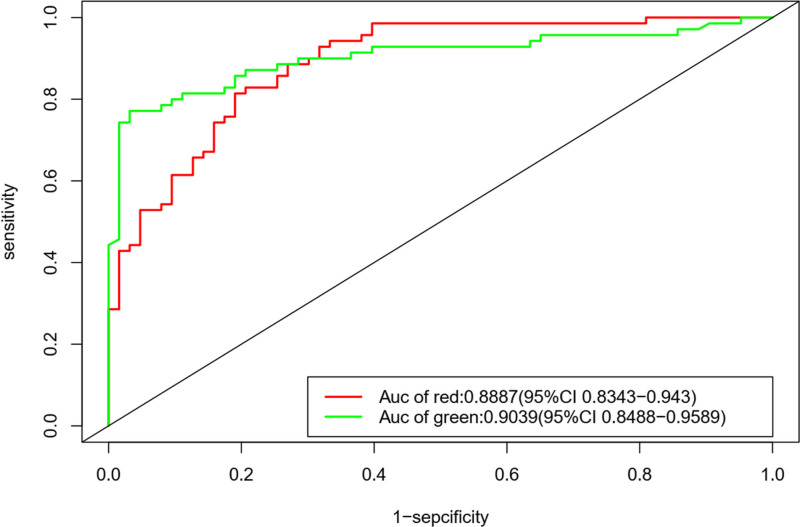
ROC curve. The ROC curves, the red curve is RPL9,and the green curve is PPP2R1A.The area under the curve is the AUC value, which is >0.75 in both cases. ROC = receiver operating characteristic.

Furthermore, ROC curves demonstrated that the ROC value for PPP2R1A was 0.9039, and for RPL9, it was 0.8887. Both ROC values exceeded 0.75, confirming the effectiveness of these 2 genes in the diagnosis of venous thrombosis (Fig. 10).

### 3.9. GSEA and GSVA of both PPP2R1A and RPL9 genes

In the GSE19151 dataset, we conducted GSEA and GSVA to elucidate pathways associated with distinct gene functions.

**Figure 11.**
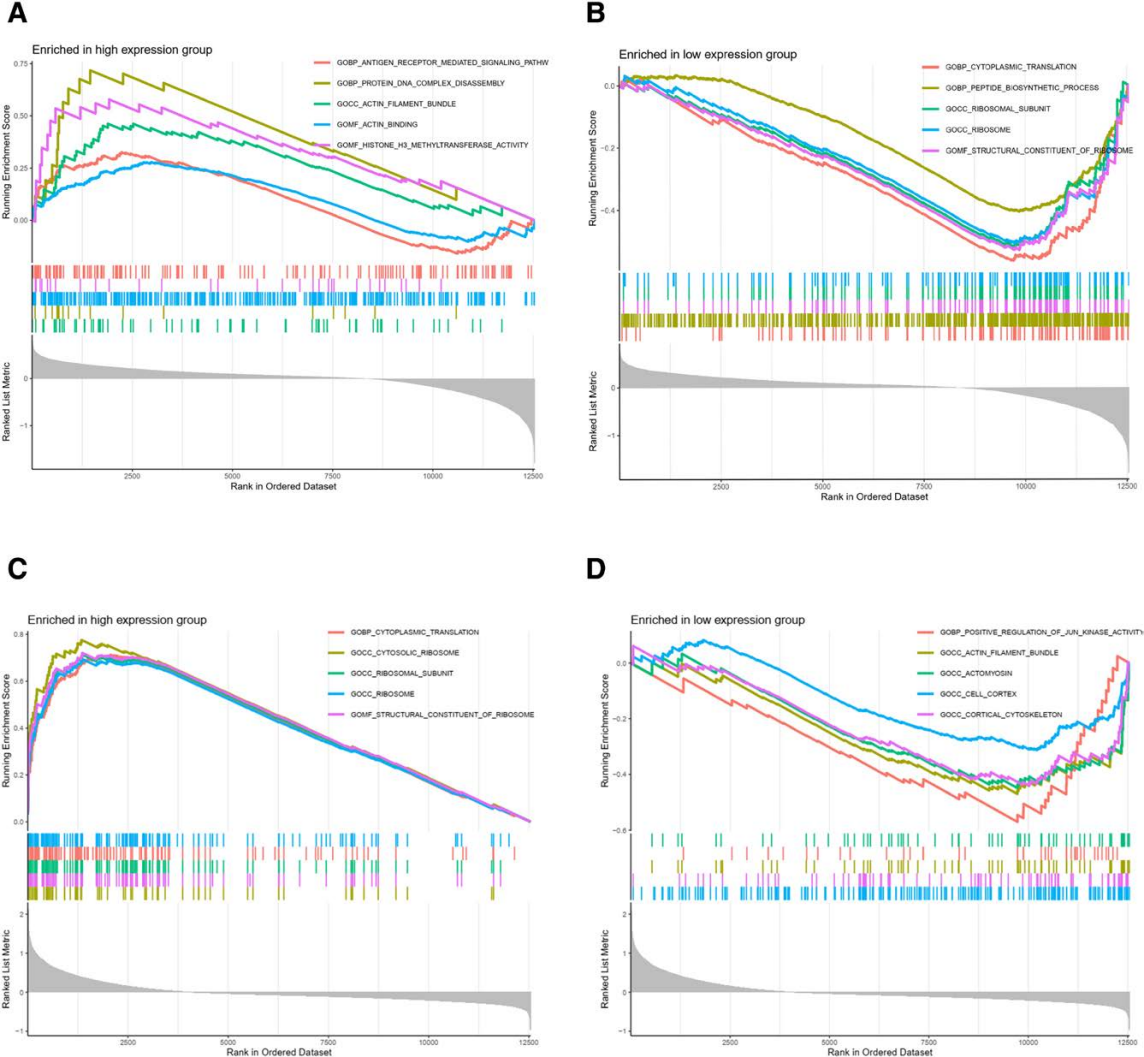
GSEA functional enrichment map. (A) The enriched graph of GSEA function score of PPP2R1A high expression group, which shows the function of antigen receptor-mediated signaling pathway, etc. (B) The enriched graph of GSEA function score of PPP2R1A low expression group, which shows the function of cytoplasmic translation, peptide biosynthesis process, etc. (C) The enriched graph of GSEA function score of RPL9 high expression group, which shows the function of cytoplasmic translation and other functions. (D) The enriched graph of GSEA function score of RPL9 low expression group, which shows the functions such as positive regulation of JUN_kinase activity. GSEA = gene set enrichment analysis.

For the PPP2R1A high-expression group, GSEA functional scoring indicated enrichment in antigen receptor-mediated signaling pathways, protein DNA complex disassembly, actin filament bundling, actin binding, and tissue H3-methyltransferase activity (Fig. 11A). Conversely, the PPP2R1A low-expression group exhibited enrichment in cytoplasmic translation, peptide biosynthetic processes, ribosomal subunits, ribosomes, and ribosomal structural components (Fig. 11B).

In the RPL9 high-expression group, GSEA functional analysis revealed enrichment in cytoplasmic translation, cytoplasmic ribosomes, ribosomal subunits, ribosomes, and ribosomal structural components (Fig. 11C). Meanwhile, the RPL9 low-expression group showed positive regulation of JUN kinase activity, actin filament bundles, actinomyosin prions, cell cortex, and cortical cytoskeleton (Fig. 11D).

**Figure 12.**
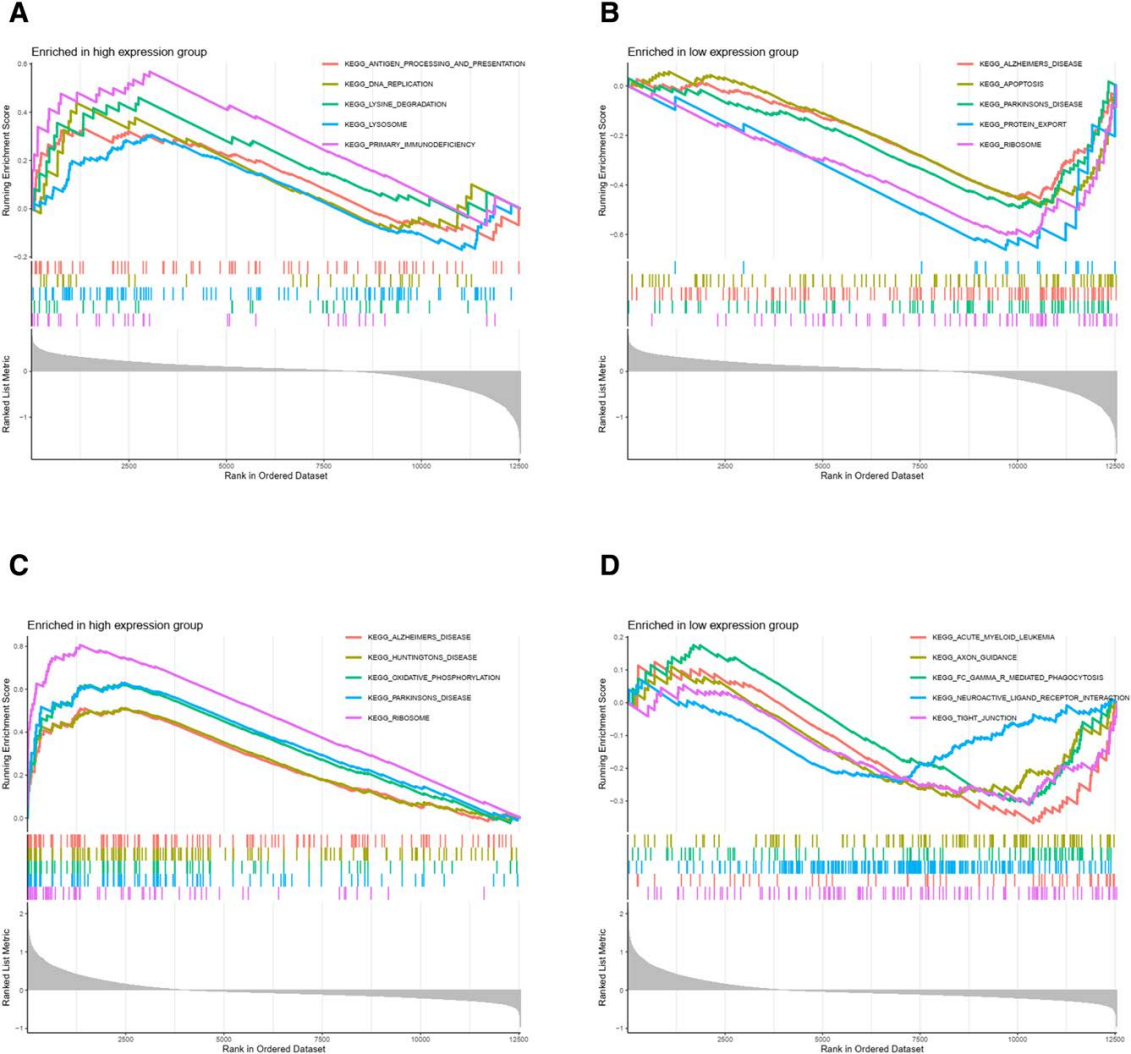
GSEA pathway enrichment map. (A) The GSEA pathway score enrichment plot of PPP2R1A high expression group, with functions such as antigen entry and presentation enriched. (B) The GSEA pathway score enrichment plot of PPP2R1A low expression group, with functions such as regulation of Alzheimer disease and apoptosis. (C) The GSEA pathway score enrichment graph of RPL9 high expression group, with oxidative phosphorylation, ribosome-related functions and so on. (D) The GSEA pathway score enrichment graph of RPL9 low expression group, with axon guidance and other functions enriched. GSEA = gene set enrichment analysis.

Regarding GSEA pathway scoring, the PPP2R1A high-expression group showed enrichment in antigen entry and presentation, lysine degradation, lysosomes, DNA replication, and primary immunodeficiency (Fig. 12A). In contrast, the PPP2R1A low-expression group exhibited enrichment in Alzheimer disease, apoptosis, Parkinson disease, protein export, and ribosomes (Fig. 12B).

For RPL9 high expression, GSEA pathway scoring highlighted enrichment in Alzheimer disease, Huntington disease, oxidative phosphorylation, Parkinson disease, and ribosomes (Fig. 12C). In the RPL9 low-expression group, enrichment was observed in acute myeloid leukemia, axon guidance, tight junctions, γ-mediated phagocytosis, and neural activity-receptor interactions (Fig. 12D).

**Figure 13.**
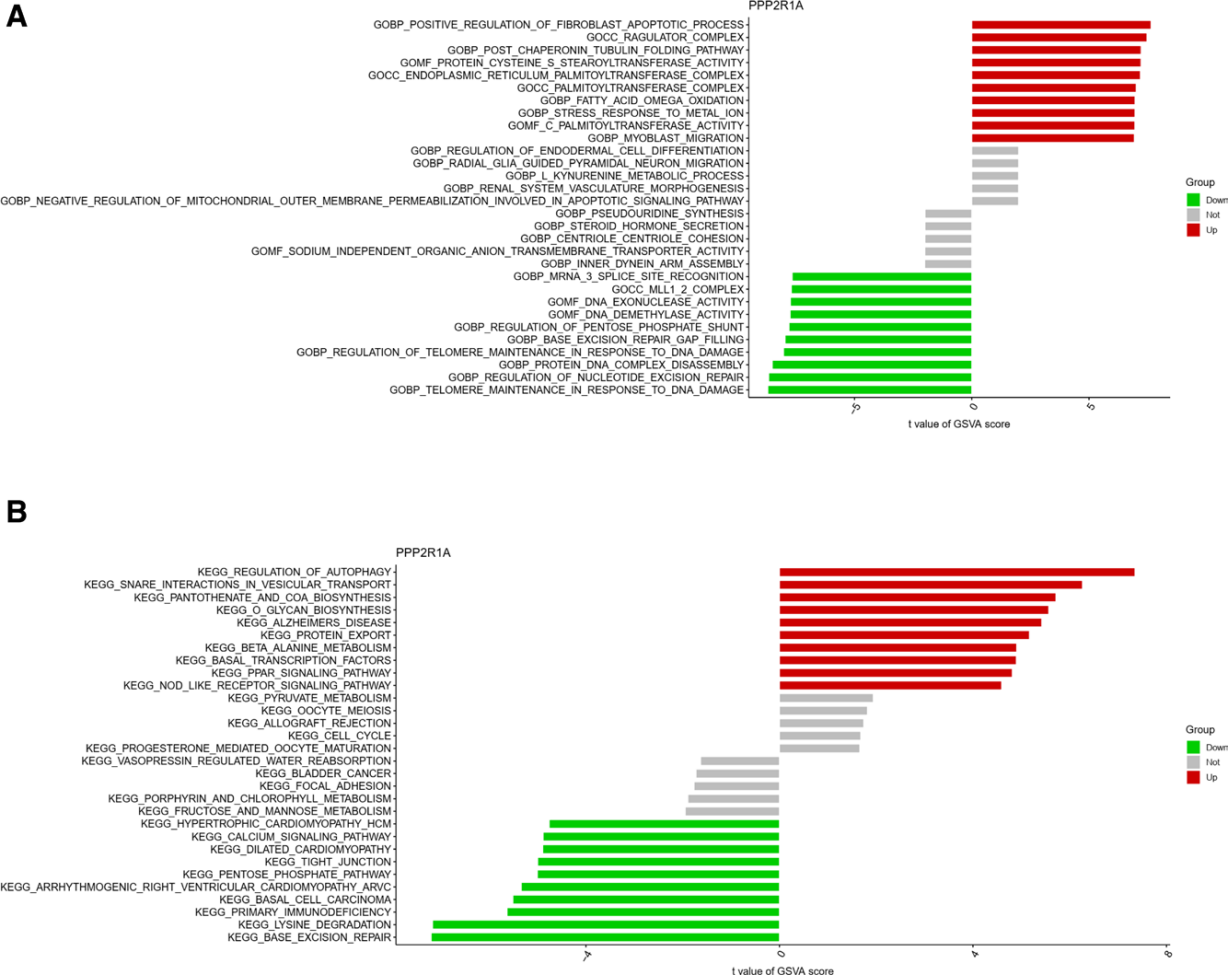
GSVA functional and pathway enrichment analysis of PPP2R1A. (A) The GSVA function enrichment analysis of PPP2R1A, which shows that there is positive regulation of fibroblast apoptosis process in the high expression group, and DNA nucleic acid exonuclease activity and other functions in the low expression group. (B) The GSVA pathway enrichment analysis of PPP2R1A, which shows that there is autophagy regulation in the high-expression group and calcium signaling pathway and other functions in the low-expression group. GSVA = gene set variation analysis.

Moving on to GSVA functional scoring, the PPP2R1A high-expression group demonstrated positive regulation of fibroblast apoptotic processes, post-chaperone protein, microtubule protein folding pathway, and ragulator_complex (Fig. 13A). Conversely, the PPP2R1A low-expression group displayed enrichment in mRNA_3_splice_site_recognition, MLL1_2_complex, DNA nucleic acid exonucleotide enzyme activity, among others (Fig. 13A).

In GSVA pathway scoring, the PPP2R1A high-expression group exhibited enrichment in autophagy regulation, trap interactions in vesicle transport, and pantothenic acid and coenzyme A biosynthesis (Fig. 13B). Conversely, the PPP2R1A low-expression group showed enrichment in fructose and mannose metabolism, hypertrophic cardiomyopathy, and calcium signaling pathway, among others (Fig. 13B).

**Figure 14.**
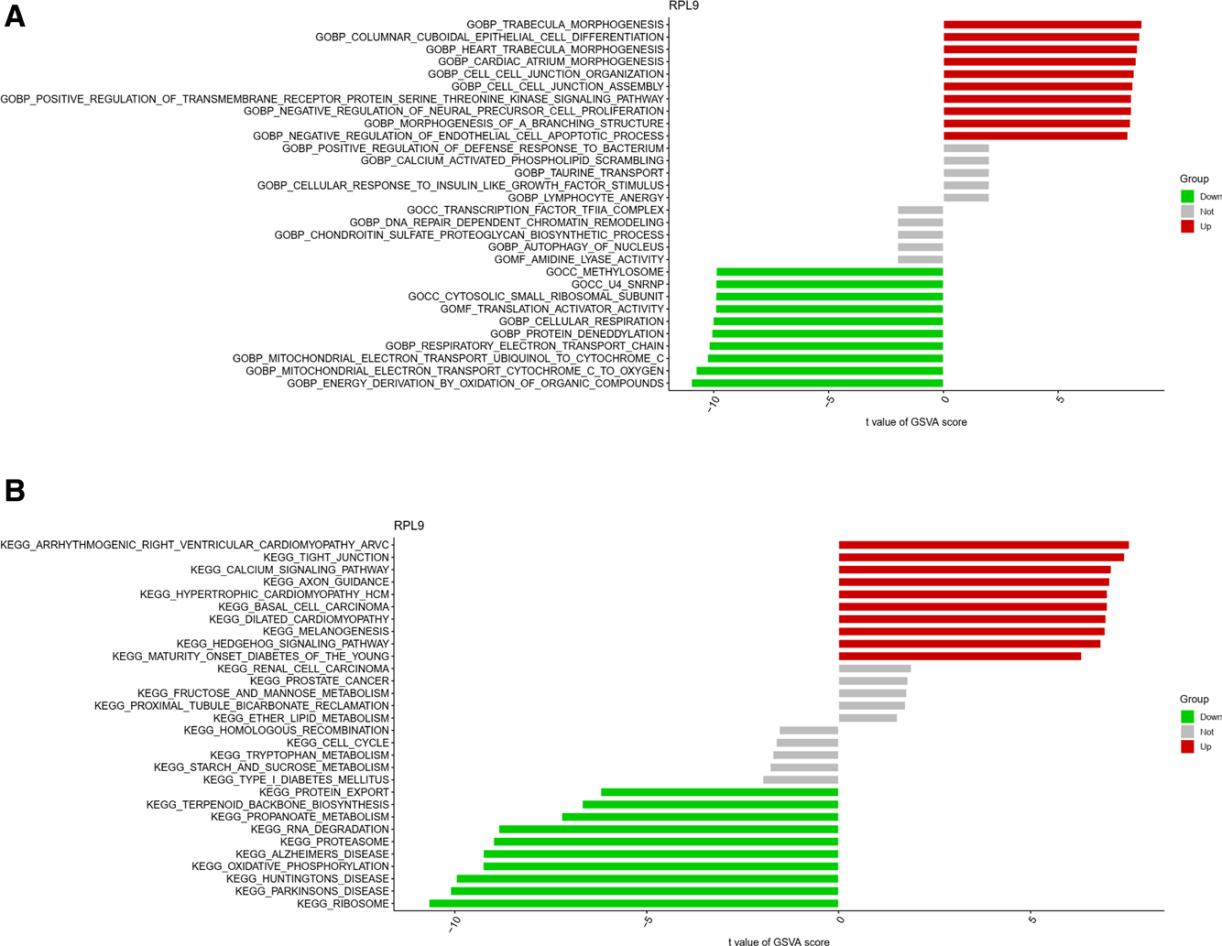
GSVA functional and pathway enrichment analysis of RPL9. (A) The GSVA functional enrichment analysis of RPL9, the high expression group has trabecular morphogenesis, and the low expression group has functions such as methyl proteins class. (B) The GSVA pathway enrichment analysis of RPL9, high expression has calcium signaling pathway, low expression has protein export and other functions. GSVA = gene set variation analysis.

Regarding GSVA functional scoring, the RPL9 high-expression group demonstrated enrichment in trabecular morphogenesis, columnar cell differentiation, and cardiopulmonary morphogenesis (Fig. 14A). In contrast, the RPL9 low-expression group displayed enrichment in methyl protein class, U4_SNRNP, and cytoplasmic small ribosomal subunit (Fig. 14A).

GSVA pathway scoring showed enrichment in arrhythmogenic right ventricular cardiomyopathy, tight junctions, and calcium signaling pathway in the RPL9 high-expression group. Meanwhile, the RPL9 low-expression group exhibited enrichment in type I diabetes, protein export, and terpene backbone biosynthesis (Fig. 14B).

### 3.10. Immune infiltration analysis

**Figure 15.**
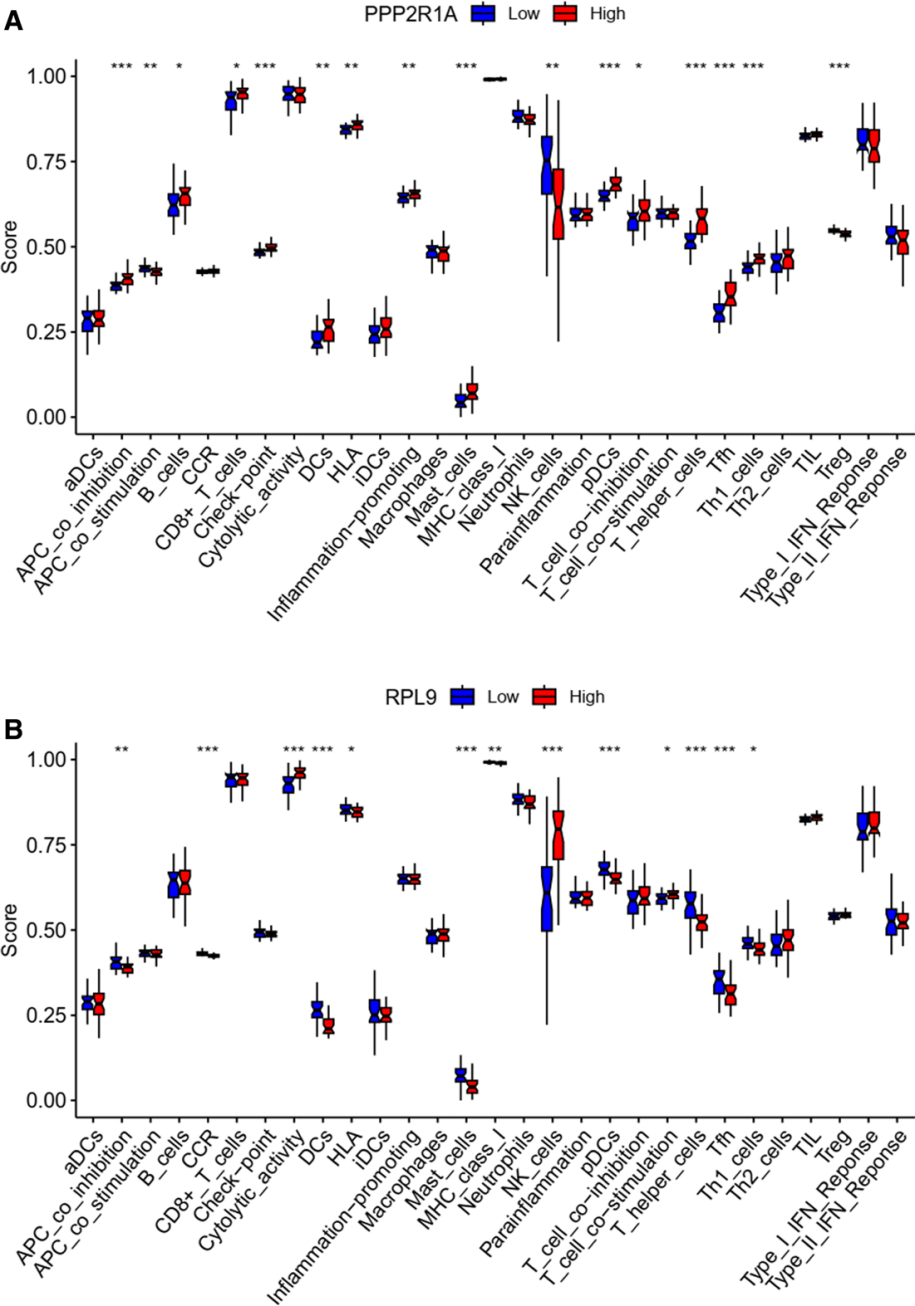
Boxplot of the difference in immune function between PPP2R1A and PRL9. (A and B) The difference in immune functions of the 2 genes, such as aDCs, APC_co_inhibition, etc are significantly different.

In our immune-related functional analysis, we identified variations in multiple immune functions between high and low expression groups of PPP2R1A and RPL9, including aDCs, APC_co_inhibition, and CD8+ T cells (Fig. 15A and B).

**Figure 16.**
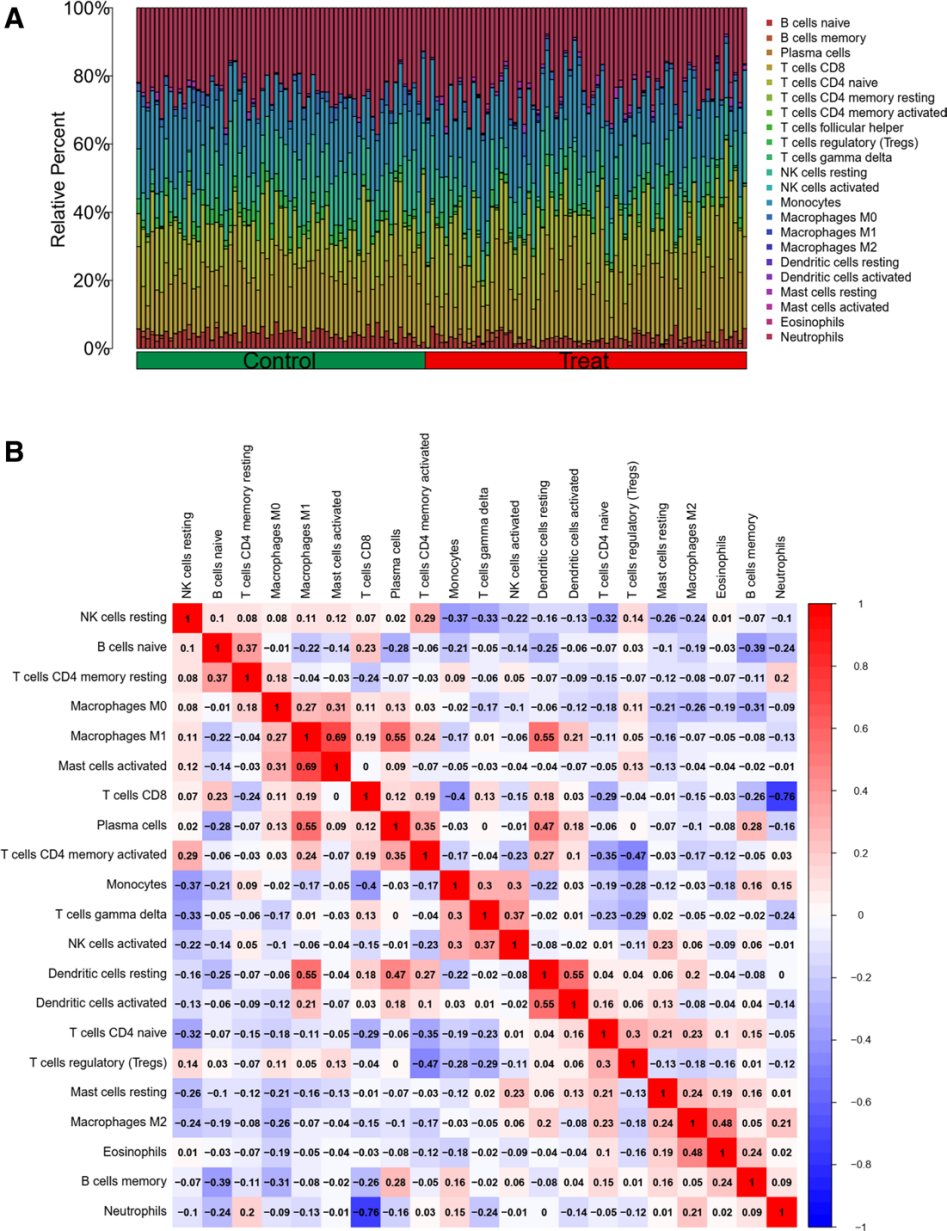
(A) The results of immune infiltration, the relative content of individual immune cells between the healthy and diseased groups. (B) The correlation of individual immune cells, with mast cells activated and macrophages M1 having the highest positive correlation and neutrophils and T cells CD8 having the highest negative correlation.

**Figure 17.**
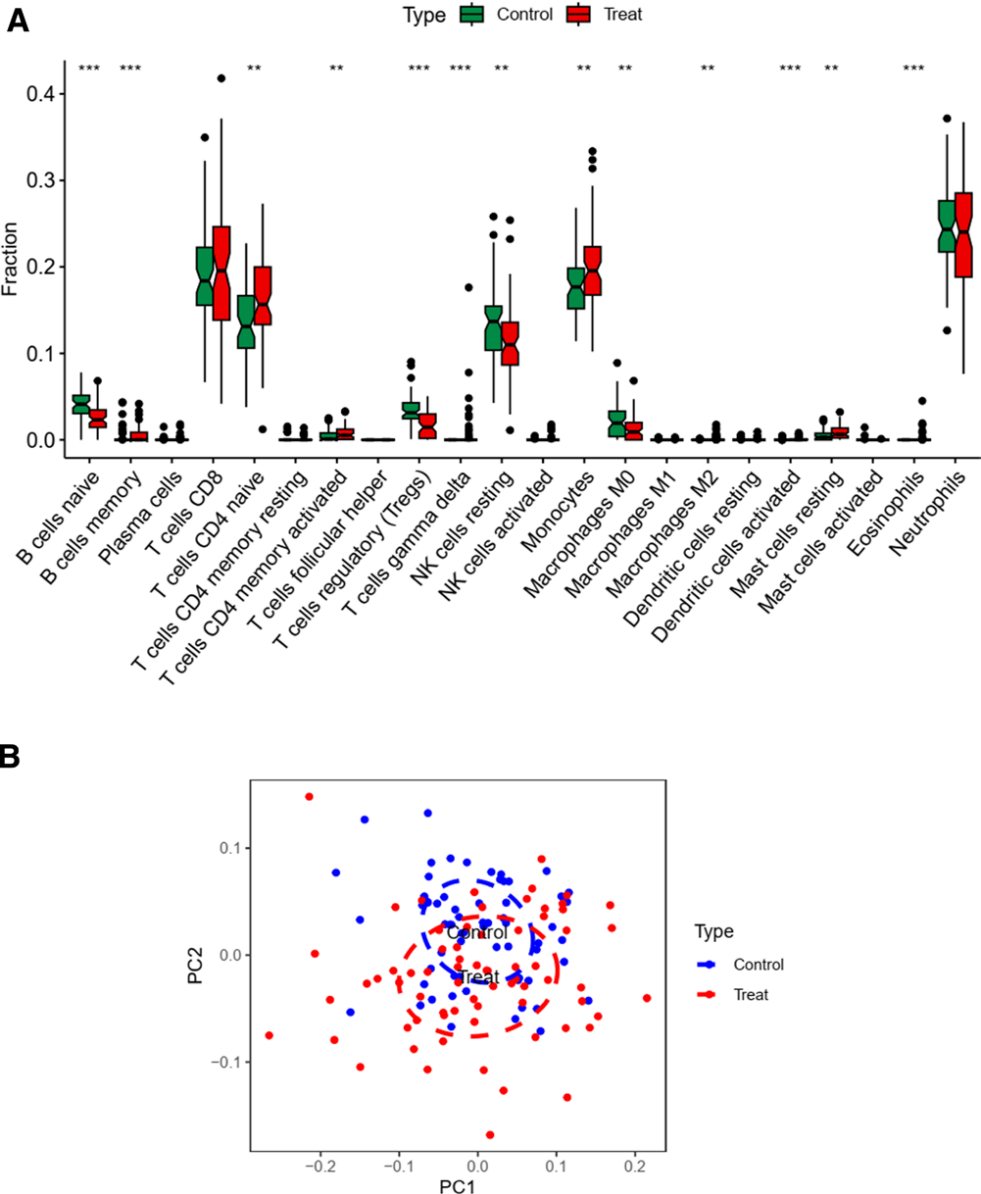
(A) The difference in expression of individual immune cells between the healthy and diseased groups, and it can be seen that most of them are differentiated. (B) The PCA plot.

To investigate immune cell infiltration, we utilized the CIBERSORT algorithm with the e1071 R package to analyze disease data and determine the relative abundance of individual immune cells (Fig. 16A). The analysis revealed the highest positive correlation between mast cells activated and macrophages M1, while the highest negative correlation was observed between neutrophils and T cells CD8 (Fig. 16B). Notably, B cells naive, B cells memory, and T cells gamma delta exhibited significance in both high and low expression groups of the disease (Fig. 17A). Principal component analysis (PCA) indicated some discernible differences between these groups (Fig. 17B).

**Figure 18.**
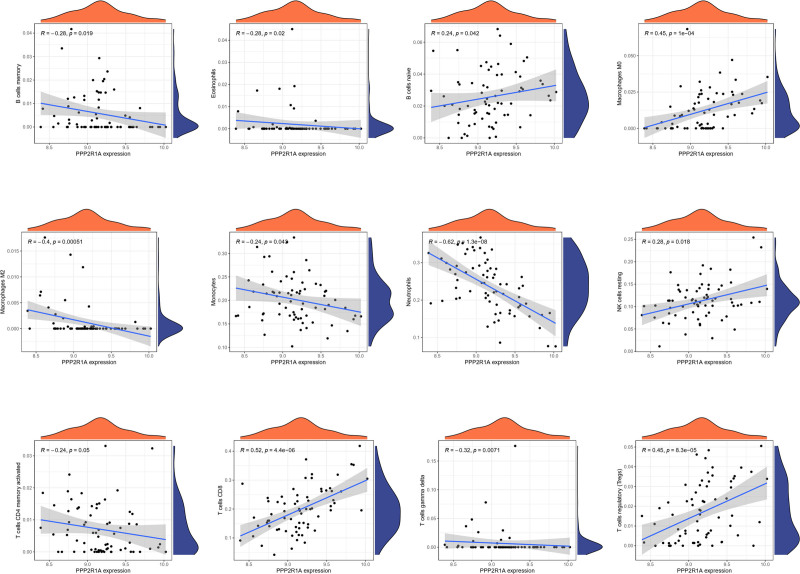
Correlation of individual differentially expressed immune cells with PPP2R1A. The correlation of individual differentially significantly expressed immune cells with PPP2R1A, with 12 more significant results.

**Figure 19.**
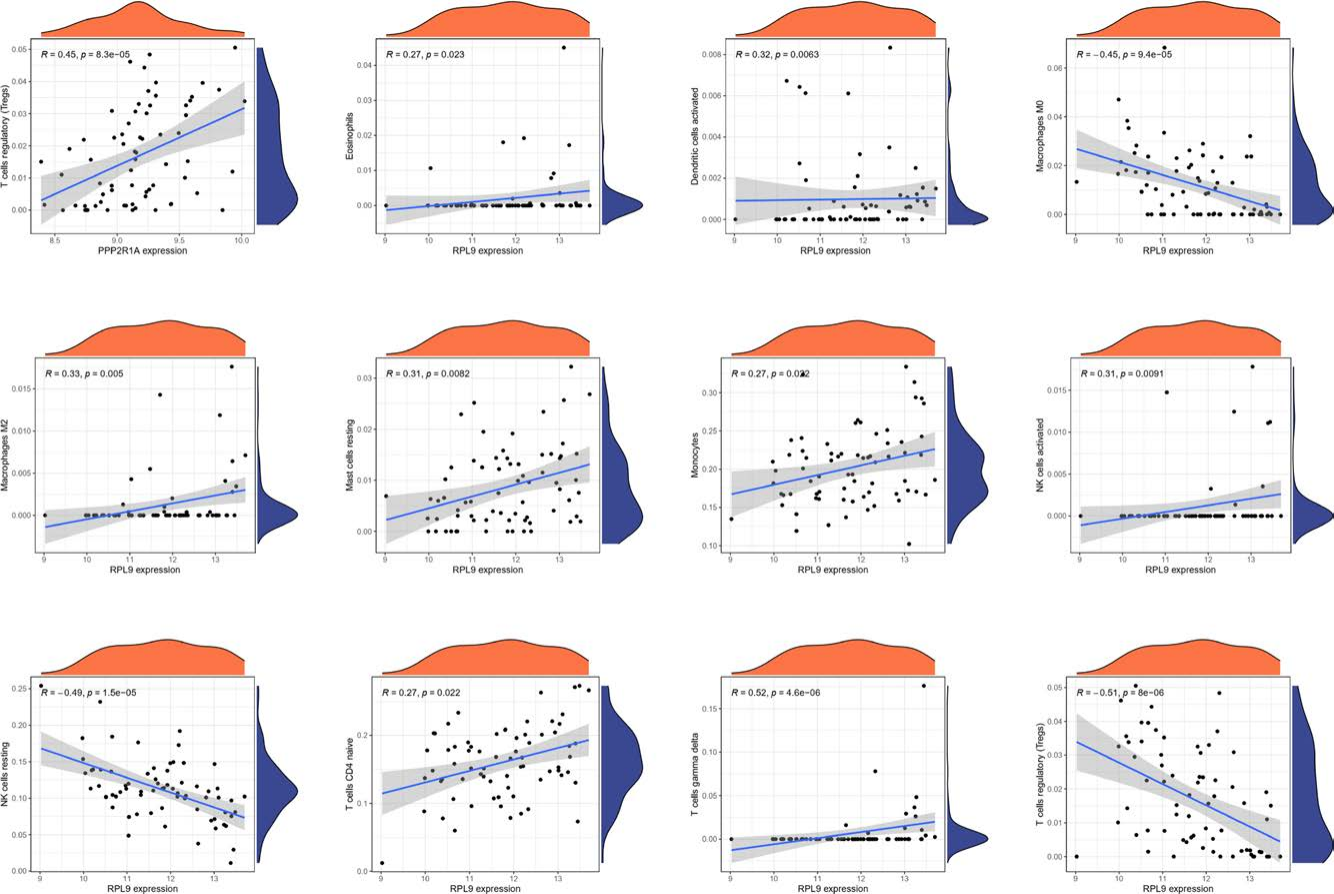
Correlation of individual differentially significantly expressed immune cells with RPL9. The correlation between individual differentially significantly expressed immune cells and RPL9, with 12 more significant results.

For PPP2R1A, immune cell correlations revealed a negative association between cor.B cells memory and its expression, while cor.B cells naive showed a positive correlation, along with 12 other immune cells (Fig. 18). Similarly, for RPL9, immune cell correlations exhibited a positive link between cor.B cells memory and its expression, as well as a positive correlation with cor.Correlation, along with correlations with 12 other immune cells (Fig. 19).

**Figure 20.**
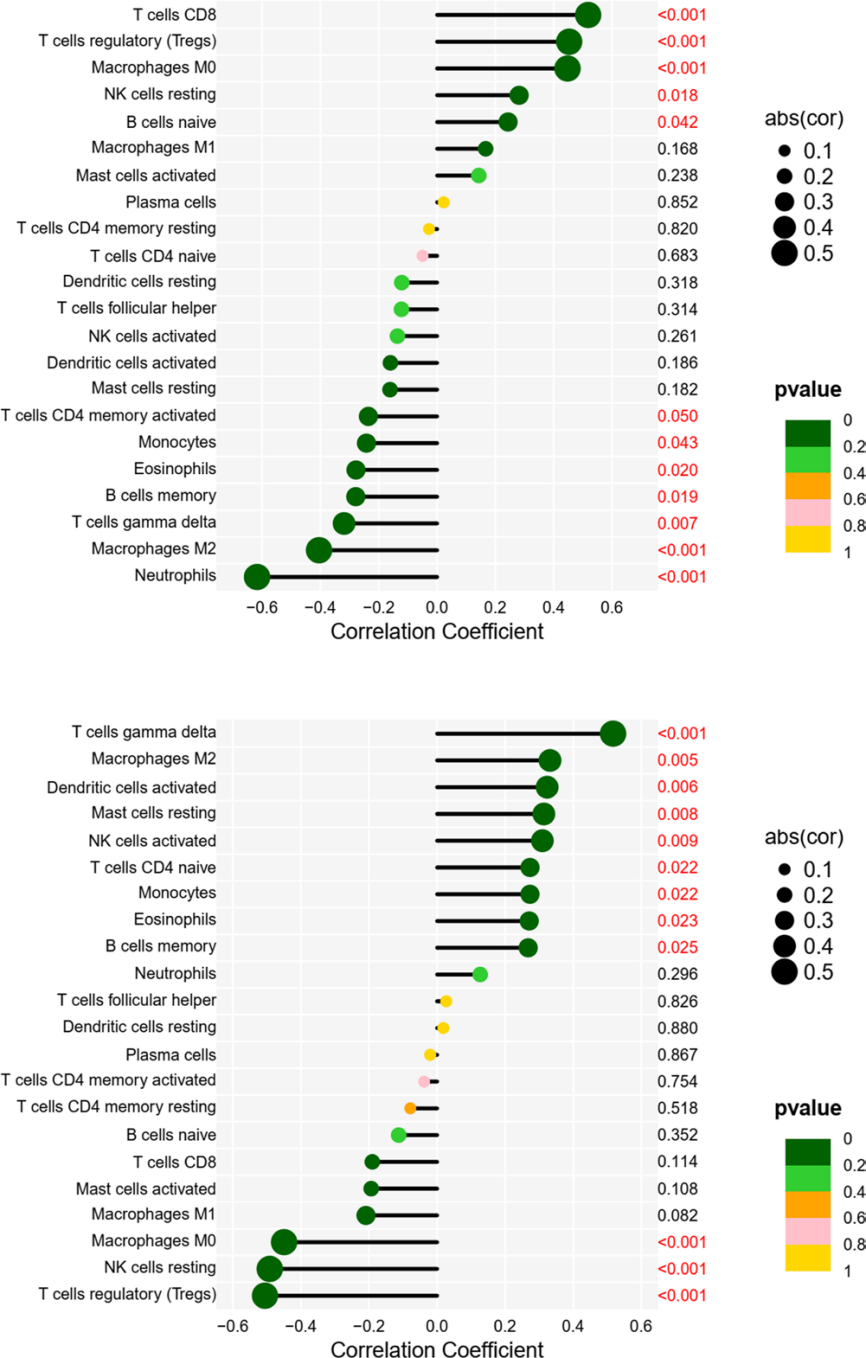
Bubble plots of PPP2R1A, RPL9, and immune cell correlation. A bubble plot of PPP2R1A, RPL9, and immune cell correlations, with immune cell names in vertical coordinates, immune correlation coefficients >0 for positive correlations and <0 for negative correlations in horizontal coordinates, and *P* values in right vertical coordinates.

Finally, we created a bubble map for visualization purposes (Fig. 20).

### 3.11. Constructing the ceRNA regulatory network map of key genes

We conducted miRNA prediction for the regulation of PPP2R1A by utilizing miRDB, miRDB, and TargetScan databases. MiRNAs predicted by all 3 databases were considered, resulting in the identification of 17 miRNAs that met the criteria. Subsequently, we predicted lncRNAs associated with these miRNAs using the spongScan database, leading to the identification of hsa-miR-625-5p and hsa-miR-15a-5p as 2 key miRNAs, along with 10 related lncRNAs.

**Figure 21.**
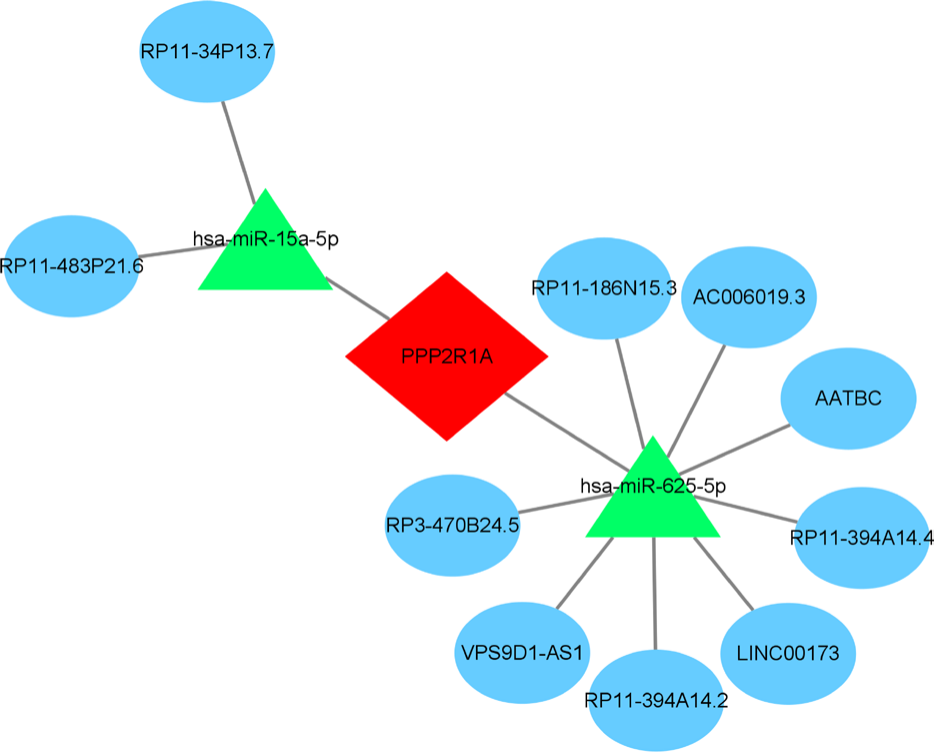
ceRNA network. The ceRNA network of PPP2R1A, with red diamonds showing that PPP2R1A is an mRNA, green triangles showing miRNAs, and blue ovals showing lncRNAs. ceRNA = competing endogenous RNA, lncRNAs = long non-coding RNAs, miRNAs = microRNAs.

According to the ceRNA hypothesis, there exists a positive regulatory relationship between lncRNA and mRNA, whereas a negative regulatory relationship exists between miRNA and mRNA. Consequently, we identified 10 lncRNAs exhibiting significant expression differences in key genes. These lncRNAs were RP11-394A14.2, AATBC, P3-470B24.5, LINC00173, AC006019.3, RP11-186N15.3, VPS9D1-AS1, RP11-483P21.6, RP11-394A14.4, and RP11-34P13.7. We then obtained node files to construct ceRNAs and proceeded to construct the ceRNA network using Cytoscape (Fig. 21).

## 4. Discussion

VTE, comprising DVT and PE, ranks among the 5 most prevalent vascular conditions worldwide.^[18]^ In the United States and Europe, conventional treatments for venous thrombosis predominantly involve intravenous anticoagulants, with newer oral drugs like rivaroxaban gaining prominence. Traditional anticoagulation therapies encompass warfarin and heparin injections, along with oral vitamin K supplements.^[19]^ From the perspective of TCM, VTE is attributed to factors such as the accumulation of dampness-heat, cold-dampness stagnation, phlegm stasis obstruction, spleen deficiency, trauma to the blood, and other elements that lead to the infiltration of dampness into the veins and collaterals. Over time, this dampness transforms into heat, causing blood stasis, impeding the flow of qi and blood, and ultimately culminating in the onset of the disease.^[20]^ DVT falls within TCM categories of “closed veins” and “swelling of femur,” with primary treatment objectives revolving around promoting blood circulation and eliminating blood stasis.^[21]^ Traditional Chinese medicines not only exhibit anti-inflammatory properties but also effectively enhance blood circulation. Recent research has revealed that active ingredients in various traditional Chinese medicines possess therapeutic potential for venous thrombosis. Consequently, the advantages of integrating traditional Chinese and Western medical approaches in treating and preventing thrombotic disorders have become increasingly apparent.^[22]^ Our study extends beyond TCM into the realm of the transcriptome, where we have identified 2 key genes. We aim to explore their impact on TCM efficacy and their association with venous thrombosis development, seeking additional therapeutic insights.

We integrated TCM principles into the treatment of VTE by selecting 5 Chinese herbs known for their properties in promoting blood circulation, removing blood stasis, and enhancing qi while addressing dampness. In our investigation, we delved into the targets and active ingredients within this compounded Chinese herbal formula (CCM) and employed a network pharmacology approach to unveil the potential targets and mechanisms of action underlying the effectiveness of CCM against VTE.

Expanding upon our network pharmacology analysis, we conducted an in-depth examination, which encompassed the clustering and typing of the constituent herbs within CCM, along with the identification of key VTE-related targets. Subsequently, we employed the WGCNA algorithm, intersecting the key genes we had screened and typed with the results derived from WGCNA analysis of VTE matrix data. Further analysis was carried out using the Lasso, Random Forest, and SVM machine learning algorithms, ultimately leading us to identify the most pivotal genes in this context: PPP2R1A and RPL9.

With PPP2R1A and RPL9 at the forefront, we further explored the ceRNA network of the PPP2R1A gene. This comprehensive approach allowed us to pinpoint the upstream and downstream regulatory miRNAs and lncRNAs, thus uncovering novel keys for the treatment of VTE. This extensive analysis represents a significant addition to the field of VTE network pharmacology, providing insights and opportunities not previously explored.

In this study, we initially identified 14 target genes associated with VTE and Cerebral Cavernous Malformation. Subsequently, regulatory networks were constructed utilizing these genes. Our analysis revealed that 4 key core genes (FOS, ICAM1, CASP3, and HSP90AA1) were consistently identified in both the PPI regulatory network and the results generated by the Cytonca algorithm. Furthermore, the network diagram depicting KEGG pathway enrichment demonstrated the significant enrichment of these 4 genes within the top 20 pathways. Chromosome circle maps illustrated the genomic locations of FOS and HSP90AA1 on chromosome 14, CASP3 on chromosome 4, and ICAM1 on chromosome 19. This observation suggests a potential shared regulatory mechanism for FOS and HSP90AA1.

The results of KEGG pathway enrichment analysis indicated a significant association between these gene enrichments and atherosclerosis. This association raises the hypothesis that these genes play a role in the regulation of atherosclerosis, which in turn impacts thrombosis. Additionally, it is suggested that the active ingredients of Chinese herbal medicine down-regulate these genes, potentially contributing to the treatment of venous thrombosis. These findings align with prior research indicating the involvement of C-FOS, the gene product of FOS, in atherosclerosis development. Moreover, alterations in C-FOS expression levels have been linked to lipid accumulation, potentially impacting thrombosis.^[23]^ ICAM1, a cell adhesion molecule, serves as an early indicator of atherosclerosis and is considered a pivotal gene for venous thrombosis treatment.^[24]^ HSP90AA1, acting as a regulatory gene, may play a crucial role in COVID-19-associated thrombosis.^[25]^ CASP3, a marker of cardiac metabolism risk factors, exhibits associations with coronary artery ion scores, potentially offering predictive evidence.^[26]^ These findings provide evidence supporting the potential utilization of herbal medicine in recurrent venous embolism treatment through gene modulation. Further investigation of the raw data involved operations like clustering and the WGCNA algorithm, along with machine learning techniques. Ultimately, we identified 2 pivotal genes, PPP2R1A and RPL9. The PPP2R1A gene encodes protein phosphatase 2A (PP2A), a crucial protein phosphatase with vital roles in cellular signaling and regulation.^[27]^ RPL9 encodes a ribosomal protein essential for ribosome assembly, a critical machinery in cellular protein synthesis.^[28]^Specifically, GSEA of the PPP2R1A gene indicated its positive regulation of processes such as protein–DNA complex disassembly, actin filament bundling, actin binding, and tissue H3-methyltransferase activity. Additionally, it exhibited negative regulation of cytoplasmic translation, peptide biosynthesis processes, ribosomal subunits, ribosomes, and ribosomal structural composition.Studies have demonstrated the indirect involvement of protein phosphatases in platelet formation and their potential role in regulating platelet function and activation states.^[29]^Consequently, we postulate that PP2A might indirectly influence platelet formation by modulating protein phosphorylation within these signaling pathways or the activity of key proteins. One plausible mechanism could involve PP2A acting as a negative regulator within specific coagulation or growth factor signaling pathways, thereby influencing cell differentiation, growth, and activation. Regarding RPL9, there is currently limited literature suggesting its direct or indirect involvement in thrombus formation. Its function and pathway enrichment primarily pertain to protein synthesis, cell cycle, cell growth and differentiation, gene regulation, etc, requiring further investigation and substantiation through additional studies.

Subsequently, we identified 12 immune cell types associated with PPP2R1A and 12 immune cell types associated with RPL9 through immune infiltration analysis. Within the context of RPL9, T cells of the gamma delta subtype exhibited the most robust positive correlation, while Tregs exhibited the most pronounced negative correlation. These correlations suggest an association between RPL9 and distinct T cell subsets involved in immune regulation, inflammation, and related biological processes.RPL9 potentially plays a role in regulating T-cell differentiation, proliferation, and immune responses. Moreover, in specific disease contexts, alterations in RPL9 expression or function could impact the abundance or activity of particular T-cell subpopulations. This suggests that RPL9 may participate in a regulatory network influencing T-cell subpopulations in collaboration with other genes and signaling molecules.Roya Ramezankhani research revealed that the RPL9 gene, housing both estrogenic and androgenic precursors, has implications in the pathogenesis of systemic immune disorders like lupus erythematosus and rheumatoid arthritis. This discovery, coupled with the observation that autoantibodies and autoreactive T cells interact with autoantigen-containing human leukocyte antigens, representing 2 pivotal facets of autoimmunity, hints at the potential relevance of RPL9 to the biological mechanisms involving T cells, including immunoregulation and inflammation.^[30]^ In the context of PPP2R1A, CD8 T cells exhibited the most significant positive correlation, while Neutrophils displayed the most pronounced negative correlation. This positive correlation suggests a potential role for PPP2R1A in the differentiation, functional regulation, or immune responses of CD8 T cells.An inverse regulatory relationship may exist between PPP2R1A and neutrophil function or regulatory mechanisms, implying that PPP2R1A could be associated with the homeostasis or regulation of various other immune cell types.An article highlights the role of PP2A as a significant regulator, not only negatively modulating IKK activity but also dephosphorylating NEMO/IKKg, thus activating the IKK complex.^[31]^ PP2A additionally exerts regulatory control over TNFα signaling through the dephosphorylation of TRAF2.^[32]^ In T cells, PP2A plays a role in augmenting cytokine production and NF-kB activity by inhibiting or down-regulating the catalytic subunit of PP2A, Cb/PPP2CB.^[33,34]^ Moreover, in activated T cells, the PP2A regulatory subunit A (PPP2R1A) directly interacts with the CBM complex, hindering the activating phosphorylation of Carma1.^[35]^ Furthermore, decreasing PP2A levels enhances the assembly of CBM complexes, leading to an increase in T cell activation.These findings establish PP2A as a negative regulator of upstream NF-kB signaling in T cells. They elucidate a causal relationship between the assembly and activity of the CBM complex and the phosphorylation level of Carma1.^[35]^

Finally, we conducted predictions of miRNAs that regulate PPP2R1A and RPL9 using the miRDB, miRDB, and TargetScan databases. Subsequently, we constructed a ceRNA network by predicting lncRNAs targeting these miRNAs via the spongScan database. However, the results for RPL9 were unsatisfactory, as no key lncRNA was identified in the predictions. In contrast, PPP2R1A exhibited more significant characteristics in the gene screening process. Therefore, we focused our efforts on predicting the ceRNA network for PPP2R1A.

In the predictions for PPP2R1A, we identified 2 key miRNAs, namely hsa-miR-625-5p and hsa-miR-15a-5p, along with 10 associated lncRNAs. Hsa-miR-15a-5p is a human microRNA known for its pivotal role in gene regulation and cellular function control.^[36]^ It has been implicated in various biological processes, including apoptosis, cell cycle regulation, and cell differentiation.^[37]^ Notably, existing literature suggests that hsa-miR-15a-5p may play a significant role in the regulation of platelet function,^[38]^ potentially offering insights into the treatment of venous embolism. Mateusz Nowicki has explored its effects on hematopoietic stem cell mobilization.^[39]^

In contrast, for hsa-miR-625-5p, there is currently no direct evidence linking it to thrombosis. However, Junbo Feng et al have suggested that it might serve as a marker for arterial entrapment, a topic warranting further investigation.^[40]^ Among the lncRNAs competing with PPP2R1A, LINC00173 stands out. Overexpression of LINC00173 has been shown to promote the proliferation and migration of vascular endothelial cells and tumorigenesis of squamous cell carcinoma cells both in vitro and in vivo. Conversely, silencing LINC00173 inhibited these processes. LINC00173 achieves this regulation by “adsorbing” miR-511-5p, leading to the upregulation of VEGFA expression. This, in turn, promotes vascular endothelial cell proliferation, migration, and the tumorigenesis of squamous cell carcinoma cells.^[41]^ This suggests the potential for LINC00173 to also modulate factors involved in thrombus formation, possibly through the sequestration of hsa-miR-625-5p, a direction we plan to explore in future investigations.

### 4.1. Advantages and limitations

This study possesses both strengths and limitations. Notably, to our knowledge, this represents one of the initial network pharmacology investigations into venous thrombosis, incorporating a machine learning methodology for key gene identification. Additionally, our study uniquely integrates immune infiltration analyses and the construction of a ceRNA network map, offering a comprehensive exploration of VTE. The data presented herein offer novel insights into the functional mechanisms underlying VTE and establish connections between active ingredient targets in TCM and disease-specific key targets. This deepens our understanding of how TCM can effectively treat VTE and provides a foundation for synergizing traditional Chinese and Western medical approaches in venous thrombosis management. Furthermore, the 2 key genes identified in our study provide valuable theoretical support for potential diagnostic and therapeutic targets.

However, certain limitations should be acknowledged. Firstly, the validation of our findings with additional datasets for key genes was not conducted. Secondly, the relatively small sample size used in our analysis may introduce errors, as it falls short of the typical sample size required for biomarker studies. Thirdly, further in vivo and in vitro experiments are imperative to confirm the roles of these 2 identified genes in venous thrombosis development. Therefore, expanding the sample size through prospective cohort studies is essential to validate our findings. Lastly, investigating the drug sensitivity of these 2 characterized genes should be an area of future exploration.

## 5. Conclusion

In summary, our study has revealed several crucial genes, including FOS, ICAM1, CASP3, and HSP90AA1, which likely play pivotal roles in the therapeutic effects of herbal medicine against venous thrombosis. Furthermore, through an in-depth analysis of their functions and downstream effects, we have identified 2 central hub genes, PPP2R1A and RPL9, which hold promise as novel targets for the treatment of VTE. Notably, these key hub genes not only show associations with thrombospondin and the regulation of platelet function but are also linked to the regulation of T cell expression within the immune system.

Additionally, our investigation into the ceRNA network centered around the gene PPP2R1A has unveiled a potential regulatory mechanism involving lncRNAs such as RP11-394A14.2, AATBC, P3-470B24.5, LINC00173, AC006019.3, RP11-186N15.3, VPS9D1-AS1, RP11-483P21.6, RP11-394A14.4, and RP11-34P13.7. These lncRNAs may compete with miRNAs (hsa-miR-625-5p, hsa-miR-15a-5p) for binding, ultimately regulating the target genes of PPP2R1A. This intricate network potentially influences the efficacy of VTE treatment.

In conclusion, while our findings are preliminary, they offer valuable insights into potential therapeutic targets for VTE. This research provides a foundation for future clinical investigations, with the aim of translating these discoveries into practical clinical applications.

## Acknowledgments

We would also like to thank Yao Xiaoxia from Lianjiang No. 3 Middle School for correcting the grammar in this article.

## Author contributions

**Conceptualization:** Ming Zhong, Tao Huang.

**Data curation:** Ming Zhong.

**Formal analysis:** Tao Huang.

**Funding acquisition:** Kaili Fu, Tao Huang, Xishi Sun.

**Investigation:** Zhuoji Li, Kaili Fu, Lingyue Song, Xishi Sun.

**Methodology:** Zhuoji Li, Ming Zhong, Xishi Sun.

**Project administration:** Zhuoji Li, Tao Huang, Dingyu Guo.

**Resources:** Zhuoji Li, Kaili Fu, Lingyue Song, Dingyu Guo.

**Software:** Zhuoji Li, Lingpin Pang, Jie Sun, Tao Huang, Lingyue Song, Dingyu Guo.

**Supervision:** Zhuoji Li, Kaili Fu, Jie Sun, Dingyu Guo, Junfen Cheng, Xishi Sun.

**Validation:** Ming Zhong, Lingpin Pang, Jie Sun, Dingyu Guo, Junfen Cheng, Xishi Sun.

**Visualization:** Ming Zhong, Jie Sun, Dingyu Guo, Junfen Cheng.

**Writing – review & editing:** Zhuoji Li, Ming Zhong.

**Writing – original draft:** Lingpin Pang.
